# Multiscale model calibrated with a Bayesian algorithm predicts long-term outcomes of treatments for mitral valve regurgitation in animal models

**DOI:** 10.1007/s10237-026-02123-0

**Published:** 2026-09-20

**Authors:** Johane H. Bracamonte, Lionel Watkins, Jeffrey J. Saucerman, Jeffrey W. Holmes

**Affiliations:** 1 Department of Biomedical Engineering, University of Alabama at Birmingham, Birmingham, AL USA; 2 Department of Mechanical and Biomedical Engineering, Boise State University, Boise, USA; 3 Department of Biomedical Engineering, University of Virginia, Charlottesville, VA USA; 4 Division of Cardiovascular Disease, Heersink School of Medicine, University of Alabama at Birmingham, Birmingham, AL USA; 5 Division of Cardiothoracic Surgery, Heersink School of Medicine, University of Alabama at Birmingham, Birmingham, AL USA

**Keywords:** Mitral regurgitation, Volume overload, Cardiac Hypertrophy, Multiscale modeling, Bayesian analysis, Machine learning, Mitral valve repair

## Abstract

In primary mitral valve regurgitation (MR), a failing valve induces volume overload (VO) of the left ventricle (LV), triggering neurohormonal responses and myocardial remodeling. Surgical valve repair is the most effective therapy for MR, improving symptoms and in some patients reversing cardiac hypertrophy. However, at least 20% of patients develop postoperative impairment of LV function with detrimental effects on long-term outcomes, with no known cause. Many MR patients receive pharmacological treatment to alleviate symptoms, with drugs that alter cardiac function, hemodynamics, and molecular pathways that influence cardiac remodeling. To support the advancement of medical management of MR, we developed a multiscale model of cardiac hypertrophy that links ventricular mechanics, cardiovascular hemodynamics, and cardiomyocyte molecular signaling. We calibrated the model using the Markov chain Monte Carlo algorithm, a machine learning method, to integrate data from 76 studies of experimental VO in animals. The model’s predictions showed good agreement with experimental data on MR in dogs, including the chronic effects of four drugs on MR-induced hypertrophy. When simulating valve repair, our model suggests that full reversal of LV hypertrophy requires both the mechanical elimination of VO and the restoration of normal neurohormonal receptor activity. Finally, we explored selecting subsets of a large pool of pre-computed simulations to personalize model predictions for a small population or individual where insufficient data are available to refit the entire model.

**Supplementary Information:**

The online version contains supplementary material available at https://doi.org/10.1007/s10237-026-02123-0.

## Introduction

Mitral valve regurgitation is a common heart disease affecting approximately 10% of people worldwide, half of whom are over 50 years of age (Wu et al. 2018). In mitral regurgitation (MR), a dysfunctional valve allows regurgitant flow from the left ventricle into the left atrium, increasing the volume the heart must pump with each beat. MR induces significant left ventricular dilation and mass increase with relatively little change in wall thickness, a pattern termed eccentric hypertrophy (Li et al. 2020; Liu et al. 2013). Depending on the extent of remodeling, this pattern of eccentric hypertrophy can evolve into dilated heart failure and death (Lorell & Carabello 2000).

Mitral valve repair is the most effective treatment for MR, producing lasting improvement of symptoms and offering the potential to reverse rather than simply arrest cardiac remodeling (Carabello 2008). However, about 20% of patients undergoing mitral valve repair develop postoperative systolic dysfunction for reasons that remain elusive (Zheng et al. 2023). Furthermore, many patients undergoing mitral valve repair are already receiving treatment with at least one drug by the time of surgery to alleviate symptoms of heart failure, and many of those drugs affect cardiovascular mechanics or hemodynamics as well as molecular signaling pathways underlying cardiac remodeling (Bishay et al. 2000; Borer & Sharma 2015; Foster et al. 2013; Le Tourneau et al. 2019). Thus, a computational model capable of predicting the combined effects of pharmacological and surgical interventions in clinical MR could improve outcomes by providing medical doctors with personalized tools to support critical decision-making. Given this long-term goal of predicting the effects of multiple treatments in combination and the fact that eccentric hypertrophy results from a complex multiscale process comprised of changes in geometry, mechanics, circulating hormones, intracellular signaling, and gene expression (Carabello 1996; Dillon et al. 2012; Oyama 2009), we sought to develop a multiscale model of eccentric hypertrophy spanning the intracellular to systems level.

Here we couple a model of left ventricular mechanics at organ scale, a circuit model of the systemic vascular circulation, and a network model of cell signaling pathways that regulate myocardial hypertrophy at molecular scale. The assembled multiscale model can simulate changes in cardiac loading, neurohormonal alterations, and drug effects by adjustment of input parameters. We employed data from 76 studies of experimental volume overload to calibrate the multiscale model using a Bayesian machine learning algorithm called Markov chain Monte Carlo (MCMC). Using the calibrated model, we generated probabilistic predictions of the range of expected left ventricular remodeling during experimental MR with and without pharmacological therapy. Our predictions showed remarkable agreement with experimental data from 47 independent studies not used for calibration, with our model-predicted 90% confidence intervals containing 97 out of 128 (76%) experimental means from untreated MR experiments. Even though the model was calibrated using data from experiments in which no drugs were administered, our model reproduced the reported effect of beta blockers (bB), angiotensin receptor antagonists (ARB), angiotensin-converting enzyme inhibitors (ACEi), and chymase inhibitors (CMAi) in the context of experimental MR. Our model also suggested a plausible mechanistic explanation for the observation that bB protects against agonist-induced hypertrophy but paradoxically exacerbates hypertrophy during MR.

These results and other simulations presented below suggest that the mechanistic multiscale model presented here—combined with a Bayesian calibration approach that integrates data from many published studies—holds promise for predicting responses to multiple interventions in combination and to interventions for which it was not specifically calibrated, as well as for better understanding the complex multiscale interactions that produce those responses.

## Methods

### Cardiovascular biomechanics model

**Fig. 1:**
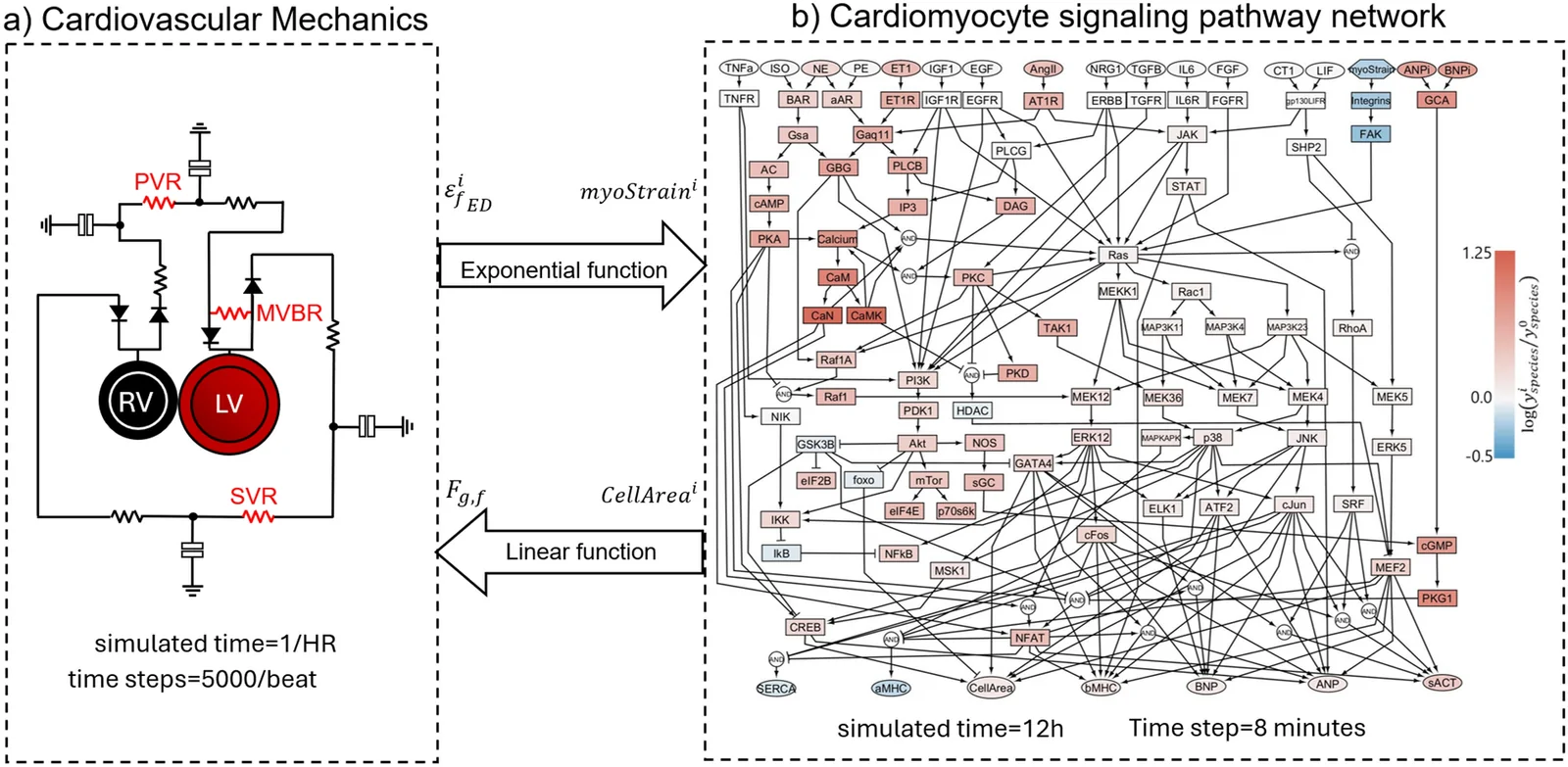
Diagram of the multiscale model consisting of **a** cardiovascular mechanics and **b** cardiomyocyte molecular signaling. **a** A thin-walled contractile sphere model of right ventricle (RV) and left ventricle (LV) mechanics coupled to a lumped model of hemodynamic circulation with adjustable systemic, pulmonary and mitral valve regurgitant flow resistance (SVR, PVR, MVBR). **b** A logic-based network model of cardiomyocyte molecular signaling relating neurohormonal receptor activity with cardiomyocyte growth. The diagram of the network model is color-coded to show the logarithm of the mean fold changes in node activity in chronic mitral regurgitation

We employed the fast-computing compartmental model of ventricular mechanics and systemic hemodynamics developed by Witzenburg and Holmes based on prior work by Burkhoff and colleagues (Santamore and Burkhoff (1991; Witzenburg and Holmes 2018). Briefly, we assumed the ventricles to be thin-walled spherical compartments with time-varying elastance such that pressure can be calculated at any moment in the cardiac cycle as,

1 $$P=\left(1-\alpha \left(t\right)\right){A}^{i}\left({e}^{{B}^{i}\left({V}_{\mathrm{E}\mathrm{D}}-{V}_{0}^{i}\right)}-1\right)+\alpha \left(t\right){E}^{i}\left({V}_{\mathrm{E}\mathrm{S}}-{V}_{0}^{i}\right)$$

where E is the end-systolic elastance of the ventricle, ${V}_{0}$ is the unloaded ventricular volume, A and B are parameters describing an exponential end-diastolic pressure–volume relationship (EDPVR), and $\alpha \left(t\right)$ is a time-dependent contractile activity function ranging from 0 (diastole) to 1 (systole). The duration of the cardiac cycle is calculated from the heart rate (HR), which is an input parameter in the model. Both ventricles shared the same EDPVR, while E for the right ventricle was set to 43% of that for the left (Santamore and Burkhoff 1991). The superindex i, indicated the time step of the growth process, meaning parameters ${A}^{i}$, ${B}^{i}$, ${E}^{i}$, and ${V}_{0}^{i}$ were growth dependent as we further elaborate in Sect. 2.4.2; we reserved superindices i=0 for the healthy baseline state and i=1 for the acute MR state. The ventricular model was coupled to a lumped (0D) model of the systemic circulation consisting of capacitors and resistors representing the systemic and pulmonary vessels, and pressure-sensitive diodes representing heart valves. Mitral regurgitation was modeled by including a resistor bypassing the mitral valve diode. The mitral valve regurgitation resistance (MVBR) was acutely dropped from infinite to a small finite value to model the abrupt onset of experimental MR and returned to infinite to model mitral valve repair or replacement (Fig. 1a). The resulting system of differential equations was solved by enforcing the conservation of the stressed blood volume (SBV) (total compartmental volume) over a cardiac cycle at a prescribed heart rate and iterating until the solution converged to a steady state. For baseline and acute MR simulations, we randomly selected values of the HR from literature-derived probability distributions. For chronic simulations, we assumed a smooth exponential transition in HR from the acute value to a value 10% above baseline over 3 months, based on the average of the data reported by Carabello et al. 1989a, b, Kleaveland et al. 1988, Matsuo et al. 1998, Nagatsu et al. 1994a, b and 2000, Nakano et al. 1990, Nemoto et al. 2002 and 2005, Pat et al. 2008 and 2010, Spinale et al. 1993, and Tsutsui et al. 1994.

The model was implemented in MATLAB to compute the pressure and volume of each compartment (left ventricle, systemic arteries, systemic veins, right ventricle, pulmonary arteries, and pulmonary veins) at 5000 time steps over the cardiac cycle (Caggiano et al. 2022; Witzenburg and Holmes 2018, 2019).

### Intracellular network model of cardiomyocyte hypertrophy

We coupled the compartmental representation of the heart and circulation to a logic-based differential equation network model of intracellular signaling pathways related to cardiac hypertrophy in myocytes (Frank et al. 2018; Ryall et al. 2012). The network model consisted of 107 nodes, 191 reactions and 17 receptor inputs (Fig. 1b). Reactions were modeled as a system of normalized Hill-type differential equations, while cross talk was modeled using continuous logical AND and OR gates. Within this normalized framework, the activity $\left(y\right)$ of each node can range from 0 (inactive) to 1 (saturated). Each reaction was characterized by three parameters: the reaction weight (w), half maximal effective concentration $(E{C}_{50})$, and Hill coefficient $({n}_{H})$. Three additional parameters modulated the activity of each node: the initial activity $\left({y}_{0}\right)$, the maximum allowed activity $({y}_{max})$, and a time constant (τ) that determines the characteristic response time (Kraeutler et al. 2010). We solved these logic-based differential equations in the MATLAB-based software Netflux available at https://github.com/saucermanlab/Netflux (Clark et al. 2024).

All network parameters were set to the default values estimated by Khalilimeybodi et al. 2020 except for the weights of the inputs (w), which were calibrated using experimental data, and the time constant (τ). The time constant for the CellArea node was set at 1140 h, the mean exponential time constant of LVM growth (T) in dogs (Bracamonte et al. 2025). We assumed the time constant τ to be 0.5% and 2% of T for intracellular reaction nodes and gene expression nodes (SERCA, aMHC, bMHC, ANP, BNP, sACT) respectively. Based on the available data from our literature review, we assumed that only the myoStrain (the node representing the mechanical stimulation of cardiomyocytes), angiotensin II (ANGII), norepinephrine (NE), endothelin 1 (ET1), atrial natriuretic peptide (ANPi), and brain natriuretic peptide (BNPi) input reactions were affected by the induction of MR, and that fold changes in concentration levels of circulating hormones would trigger proportional changes in the hormone receptor reaction input in the cardiomyocyte signaling model. The network model in a format compatible with Netflux is provided as Online Resource 1.

### Data collection

We collected data from 54 research articles on experimental mitral regurgitation in dogs and 33 articles on experimental volume overload (VO) by aortocaval shunt in rats. All data were reported as a mean value and standard deviation (SD), and we assumed that the underlying experimental measurements followed a normal probability distribution function (PDF). We utilized hormonal and molecular data from both species to calibrate and validate the cardiomyocyte signaling pathway model, as reported elsewhere (Bracamonte et al. 2025). These data included circulating levels of hormones relevant to hypertrophic signaling (AngII, NE, ET1, NE, ANPi, and BNPi), and activation/phosphorylation levels of signaling proteins focal adhesion kinase (FAK), Akt, ERK5, ERK12, ELK1, cGMP, p38, and JNK. For calibration and validation of the left ventricular mechanics and circulation models, we used only canine data. These data included left ventricular mass $\left(LVM\right)$, left ventricular end-diastolic volume and pressure $\left( {V_{{{\mathrm{ED}}}} ,~~P_{{{\mathrm{ED}}}} } \right)$, left ventricular end-systolic volume and pressure $\left( {V_{{{\mathrm{ES}}}} ,~~P_{{{\mathrm{ES}}}} } \right)$, left ventricular end-diastolic free-wall thickness $\left( {h_{{{\mathrm{ED}}}} } \right)$, left ventricular maximum rate of pressure change $\left(\frac{dP}{d\mathrm{t}}\right)$, arterial mean blood pressure $\left(MAP\right)$, arterial systolic $\left(SBP\right)$ and diastolic blood pressure $\left(DBP\right)$, mean pulmonary artery pressure $\left(MPAP\right)$, heart rate $\left(HR\right)$, stroke volume $\left(SV\right)$, ejection fraction (EF), systemic vascular resistance $\left(SVR\right)$, and pulmonary vascular resistance (PVR). We provide a detailed list of all data sources and references in Online Resource 2.

### Coupling of the multiscale model

Considering that filling and ejection during each cardiac cycle occur over tenths of seconds while muscle growth requires hours to be measurable at macroscopic scale, we solved the biomechanical and the signaling models independently for each simulated growth step. On each growth step (i), we simulated one cardiac cycle (~ 1 s) to calculate hemodynamics and biomechanical parameters, including the mechanical stimulus for growth. To simulate a single cardiac cycle, we iterated multiple times through all time steps before the biomechanical model converged to a steady state, after which further iterations produced minimal changes in the estimated pressures, volumes, and flows within that one cardiac cycle. We then used the steady-state converged biomechanics to calculate the mechanical strain and update the myoStrain network input. Then, we applied the mechanical and neurohormonal inputs to the network model and simulated 12 h of myocardial growth. The estimated growth was then communicated back to the mechanical model to update ventricular dimensions and properties for the next growth step (Fig. 1). The communication between the biomechanics and the signaling network model employed two mapping functions: one converting the organ-scale ventricular strain into the normalized network input myoStrain and a second mapping the network model-predicted value of CellArea into changes in ventricular growth.

#### Mapping myocardial strains to the network normalized myoStrain network input

It is known that cardiomyocytes can adapt their size, shape, and orientation in response to altered biomechanical stimuli. This phenomenon has been modeled assuming that either mechanical stress or deformation are the drivers of this adaptive response (Sharifi et al. 2021). Strain-driven growth models have successfully reproduced growth responses to pressure overload, volume overload, myocardial infarction, and postnatal development (Holmes 2004; Yoshida & Holmes 2021). Following some of those prior studies, we used maximum fiber strain ${\varepsilon}_{{f}_{\mathrm{E}\mathrm{D}}}$, which occurs at end diastole within the cardiac cycle as the mechanical driver of eccentric ventricular hypertrophy (Kerckhoffs et al. 2012; Witzenburg & Holmes 2017). At any given time step i, maximum fiber strain can be calculated on a thin-walled spherical shell as:

2 $${\varepsilon}_{{\mathrm{f}}_{\mathrm{E}\mathrm{D}}}^{i}=\frac{1}{2}\left[{\left({V}_{\mathrm{E}\mathrm{D}}^{i}/{V}_{0}^{i}\right)}^\frac{2}{3} -1\right]$$

where ${V}_{\mathrm{E}\mathrm{D}}^{i}$ and ${V}_{0}^{i}$ are the end-diastolic and unloaded volume of the left ventricle at the simulated growth step *i*. In the cell signaling model, the relationship between CellArea (representing the hypertrophic response) and myoStrain is a sigmoid function that has its maximum slope at a myoStrain of 0.055 and saturates at a myoStrain value of 0.21. We therefore defined an exponential mapping function so that it would convert typical fiber strain values from the baseline model to a myoStrain value of 0.055 and typical fiber strains during the most severe simulated cases of acute MR to a myoStrain value of 0.21. These selections were made with the intention of maximizing the sensitivity of the hypertrophic response to changes in mechanical stimulation:

3 $$w_{{{\mathrm{myo}}\,{\mathrm{Strain}}}} = 0.01\left( {e^{{1.8718\varepsilon_{{f{\mathrm{ED}}}}^{i} /\varepsilon_{{f{\mathrm{ED}}}}^{0} }} - 1} \right),$$

#### Mapping cardiomyocyte growth to organ-scale growth

We adopted the kinematic growth framework by (Rodriguez et al. 1994), and implemented it as in previous publications using similar compartmental models of hypertrophy (Witzenburg and Holmes 2018; Yoshida et al. 2022). Briefly, the deformation gradient ${\boldsymbol{F}}$ was decomposed into elastic stretch $\left({{\boldsymbol{F}}}_{{\boldsymbol{e}}}\right)$ and growth stretch $\left({{\boldsymbol{F}}}_{{\boldsymbol{g}}}\right)$ tensors:

4 $${\boldsymbol{F}}={{\boldsymbol{F}}}_{{\boldsymbol{e}}}\bullet {{\boldsymbol{F}}}_{{\boldsymbol{g}}},$$

where ${{\boldsymbol{F}}}_{{\boldsymbol{g}}}$ is a diagonal tensor and its components represent the growth in fiber $\left({F}_{g,f}\right)$, radial $\left({F}_{g,r}\right)$ and cross-fiber $\left({F}_{g,c}\right)$ orthogonal directions. In this model of eccentric hypertrophy, we assumed all growth occurs in the fiber direction, with no growth in radial nor cross-fiber directions $({F}_{g,r}={F}_{g,c}=1)$. This growth model was coupled to the signaling network for myocyte hypertrophy by linearly mapping fold changes in activity of the CellArea node $\left( {y_{{{\mathrm{CellArea}}}}^{i} /y_{{{\mathrm{CellArea}}}}^{0} } \right)$ onto the fiber direction component of the growth tensor $\left({F}_{g,f}\right)$:

5 $${F}_{g,f}=0.58\frac{{y}_{\mathrm{C}\mathrm{e}\mathrm{l}\mathrm{l}\mathrm{A}\mathrm{r}\mathrm{e}\mathrm{a}}^{i}}{{y}_{\mathrm{C}\mathrm{e}\mathrm{l}\mathrm{l}\mathrm{A}\mathrm{r}\mathrm{e}\mathrm{a}}^{0}}+0.42$$

The constants of this mapping function were adjusted so that the mean fold change in CellArea translated into a *1.37*-fold change in LV mass, the expected chronic mean growth in dogs. For a thin-walled sphere, the unloaded LV dimensions can be written as a function of the corresponding growth tensor component and the unloaded configuration at baseline,

6 $${V}_{0}^{i}=\frac{4\pi }{3}{\left({F}_{g,f}^{i} {r}_{0}^{0}\right)}^{3},$$

where ${r}_{0}$ is the radius of the unloaded sphere (of volume ${V}_{0}$) and superindex i indicates the growth time step, with i=0 reserved for baseline. We assumed that growth does not affect the intrinsic passive and active material properties of the myocardium, meaning that the myocardial end-diastolic and end-systolic stress–strain relationships remain constant. However, even if the intrinsic stress–strain properties were unchanged, the left ventricular pressure–volume relationships and related parameters ${A}^{i}$,${B}^{i}$, ${V}_{0}^{i}$, and ${E}^{i}$ do change with LV size. At any given growth step i, the pressure–volume parameters can be written as:

7 $${A}^{i}={a}^{0}\frac{3}{4\pi }{\left({r}_{0}{F}_{g,f}^{i}\right)}^{-3}$$

8 $${B}^{i}=2{b}^{0}\frac{{h}_{0}{F}_{g,r}^{i}}{{r}_{0}{F}_{g,f }^{i}}$$

9 $${E}^{i}={e}^{i}\frac{3}{2\pi }\frac{{h}_{0}{F}_{g,r}^{i}}{{\left({r}_{0}{F}_{g,f }^{i}\right)}^{4}}$$

where ${a}^{0}$ and ${b}^{0}$ represent intrinsic passive mechanical properties of the myocardium and ${e}^{i}$ is a representation of the intrinsic contractility of the myocardium (Witzenburg and Holmes 2018).

#### Representing reflex-mediated changes in myocardial contractility

One important reflex-mediated response to volume overload is the catecholamine-induced augmentation of intrinsic myocardial contractility. This compensation mechanism was observed by Nagatsu et al. (1994a, b), who reported that dogs with severe experimental MR maintained apparently normal pump function for 3 months but displayed systolic dysfunction under the effects of beta blockers, confirming that the adrenergic system was compensating systolic dysfunction by increasing contractility (Nagatsu et al. 1994a, b). To explore the implications of such compensation on the cardiac remodeling process, we considered two scenarios: a fully compensated case, where intrinsic contractility increased as much as needed ${(e}^{i}>{e}^{0})$ to hold the chamber elastance constant at its baseline value $\left({E}^{i}={E}^{0}\right)$, and complete elimination of compensation due to beta adrenergic blockade, where intrinsic contractility was set at baseline $\left({e}^{i}={e}^{0}\right)$, and chamber-level elastance consequently decreased with eccentric growth (Eq. 9).

### Calibration of the multiscale model

To manage the number of parameters to be calibrated simultaneously, the cardiovascular biomechanics model was calibrated independently from the cardiomyocyte hypertrophy model. No single experiment reported in the literature provides enough information to fully calibrate either model, so we implemented a Bayesian inference framework to estimate the probability distribution functions for key model parameters based on data from multiple studies. The Bayesian machine learning tool utilized for this study was a standard Markov chain Monte Carlo (MCMC) algorithm (Andrieu et al. 2003), with Gibbs sampling for the navigation of the multiparametric space, and a Metropolis–Hastings selection criteria with symmetric proposal function (Carlin and Chib 1995; Hastings 1970), to determine the space of parameter sets that most likely describe the target experimental data (Dhamala et al. 2018; Efendiev et al. 2006).

To identify the burn-in phase and check the convergence of the MCMC, we calculated mean and standard deviation of all calibrated variables every 5000 iterations. We identified the burn-in phase as the collection of initial iterations where the 5000-iteration statistics showed evident change. We considered the MCMC to be converged when the difference in mean and standard deviation between two consecutive measures fell below 1% for all calibrated parameters. Additionally, we verified convergence using cumulative means and standard deviation calculated from the end of the burn-in phase. More details about the Markov chain Monte Carlo methods, computational implementation, and performance metrics are provided in Online Resource 3.

#### Calibration of the cardiovascular biomechanics model

**Fig. 2:**
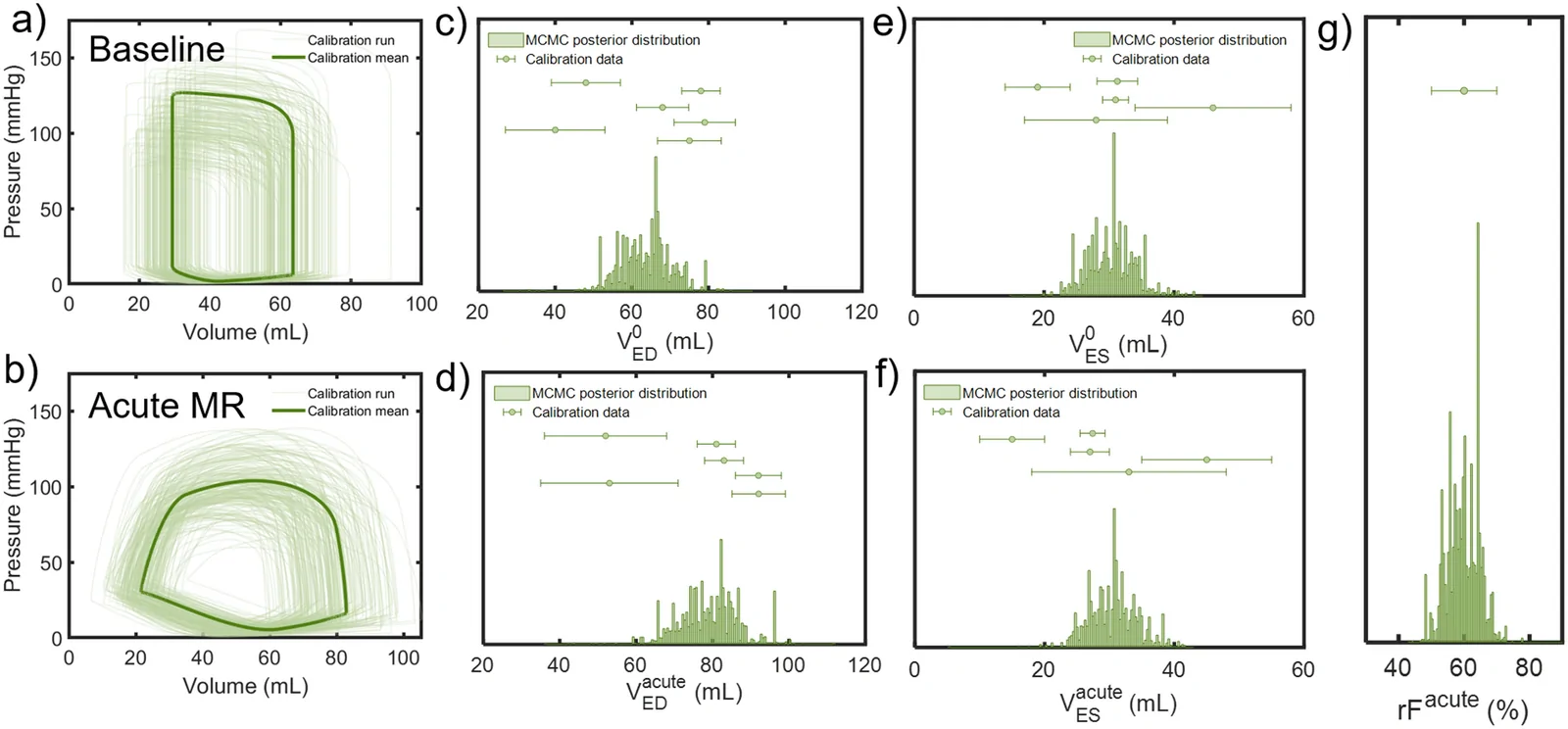
Markov chain Monte Carlo (MCMC) calibration of the cardiovascular mechanics model using canine data from baseline and acute MR. Pressure–volume loops of selected MCMC iterations are shown for **a** baseline and **b** acute MR. Converged MCMC posterior probability distribution functions (PDF) (histogram) are compared to experimental data used for calibration (markers) for **c** healthy baseline and **d** acute MR end-diastolic volume *V*_ED_, and **e** healthy baseline and **f** acute MR end-systolic volume *V*_ES_. **g** Acute regurgitant fraction posterior PDF versus targeted distribution of 60±10%

Similar to Witzenburg and Holmes (2018), we calibrated the mechanical model based on hemodynamics from the healthy baseline and acute MR states, allowing the calibration of this component of the model without the complications introduced by growth (Fig. 2). We set all parameters for the vasculature circulation model at the values employed by Santamore and Burkhoff (1991), except for the stressed blood volume $\left(SBV\right)$, systemic vascular resistance (SVR), pulmonary vascular resistance (PVR), and mitral valve regurgitant flow resistance (MVBR). These, along with the ventricular parameters ${A}^{0}, {B}^{0}, {E}^{0}, {V}_{0}^{0}$ were calibrated to experimental data. Ventricular parameters $({A}^{0}, {B}^{0}, {E}^{0}, {V}_{0}^{0})$ were assumed to be the same immediately before and after the onset of regurgitation, while the circulation parameters ($SV{R}^{0},$ $PV{R}^{0}, SB{V}^{0},MVBR^{0}$) are allowed to change acutely ($SV{R}^{1},$ $PV{R}^{1}, SB{V}^{1},MVBR^{1}$) after experimental MR, adding up to 11 unknown parameters since $MVBR^0$ is infinite.

The experimental data used for calibration of the mechanical model were collected from 8 experimental MR studies that reported measurements before and within 96 h after surgical chordae tendineae rupture in dogs (Berko et al. 1987; Carabello et al. 1989b; Imamura et al. 1994; Katayama et al. 1988; Kleaveland et al. 1988; Nakano et al. 1991; Prasad et al. 1977; Tsutsui et al. 1994), and 4 studies reporting myocardial end-diastolic stretch in healthy dog hearts measured from an unloaded configuration (Fomovsky et al. 2012; Ross et al. 1971; Ross and McCullagh 1972; Spotnitz et al. 1966). The data included baseline and acute MR measurements of ${V}_{ED}$, ${V}_{ES}$, ${P}_{ED}$, ${P}_{ES}$, $dP/d{t}_{max}$, MAP, DBP, MPAP, EF, and the baseline end-diastolic fiber stretch ${\lambda}_{ED}^{0}$, for a total of 43 experimental datasets. We included an acute regurgitant fraction (rF) of 60±10% as an additional data point when computing the likelihood of parameter sets during calibration (Fig. 2g), to enforce severe simulated MR similar to that reported in the calibration studies (Carabello et al. 1989a; Imamura et al. 1994; Kleaveland et al. 1988; Tsutsui et al. 1994; Urabe et al. 1992).

#### Calibration of the Intracellular network model of cardiomyocyte hypertrophy.

In the network model, the reaction input weights (w) determine the relative sensitivity of the network to changes in the respective biochemical or mechanical inputs. We estimated the PDFs of these weights at baseline using the MCMC method as described in detail elsewhere (Bracamonte et al. 2025). We fixed the reaction weight of the mechanical stimulation input (myoStrain) at 0.055 and calibrated the baseline weight of the AngII, NE, and ET1 and the background reaction weight assigned to the rest of the inputs. We define background reactions as any network input not known to be strongly affected by mitral regurgitation; these inputs remained at their basal level of activation throughout the simulations. In a previous study, we found that the CellArea node was insensitive to the reaction weight of inputs ANPi and BNPi, and we therefore assigned them the same weight as the background reactions in the work presented here (Bracamonte et al. 2025).

On each calibration run, we randomly imposed data-derived time-varying curves for the mechanical and neurohormonal inputs, while the likelihood of the outputs was evaluated against data on intracellular protein activity and changes in cell area from dog and rat experiments of volume overload. To integrate data from different animal species along the timeline of volume overload, we normalized the time scale by the time constant of an exponential growth curve (T) fitted to LVM data from each species.

**Fig. 3:**
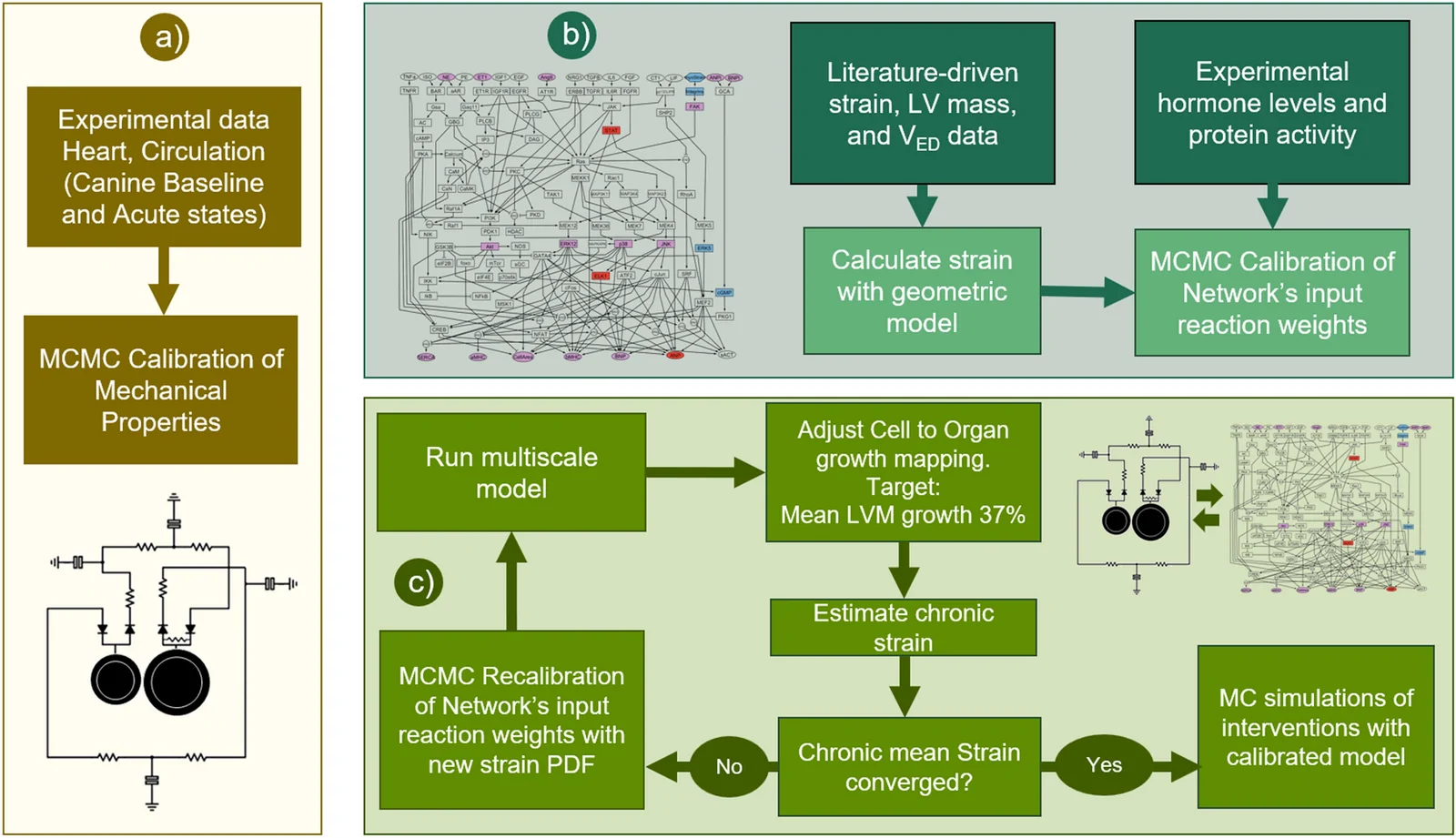
Calibration of the coupled multiscale model. **a** The cardiovascular mechanics model was calibrated independently from the network model by integrating data from baseline and acute MR states where LV growth was negligible. **b** We performed a preliminary calibration of the network model using a data-driven geometric estimation of ventricular strain and experimental data from VO experiments in dogs and rats, as described in Bracamonte et al. (2025). **c** We iteratively recalibrated the coupled network model by updating a multiscale model-generated strain input by simulating untreated experimental MR in dogs while targeting a mean LV mass increment of 37%

Because the calibration of the network model depends on a mechanical stimulation input mapped from the cardiovascular mechanics model, we relied on an iterative process. On the first iteration the time-varying curves of end-diastolic strain were estimated from mass and volume data as in a previous work (Bracamonte et al. 2025). Once the network was calibrated, we assembled the full multiscale model and generated new time-varying end-diastolic strain curves by simulating untreated MR, which then were used to recalibrate the network component (Fig. 3). The multiscale model-generated PDFs of end-diastolic strain converged after just two iterations.

### Simulations of untreated MR and interventions

Smooth Gaussian functions were fitted to the MCMC-derived posterior PDFs of all mechanical and network parameters. Model predictions were performed with Monte Carlo (MC) simulations generated by randomly sampling the smoothed PDFs of mechanical input parameters (${A}^{0}$, ${B}^{0}$, ${E}^{0}$, ${h}_{ED}^{0}$, $SV{R}^{0}$, $SV{R}^{1}$, $PV{R}^{0}$, $PV{R}^{1}$, $SB{V}^{0}$, $SB{V}^{1}$, $MVBR^1$, ${HR}^{0}$, ${HR}^{1}$) and network model input reaction weights (${w}_{ANGII}^{0}$, ${w}_{NE}^{0}$, ${w}_{ET1}^{0}$, ${w}_{back}^{0}$). After examining the covariance matrix of input parameters resulting from the MCMC calibration, we decided to sample all input variables independently except for ${A}^{0}$ and ${B}^{0}$ (Online Resource 4), ${HR}^{0}$ and ${SVR}^{0}$, and ${HR}^{1}$ and ${SVR}^{1}$ pairs (Online Resource 3 Section ESM 3.3). These dependent pairs were sampled from a Gaussian bivariate function fitted to the outputs of the MCMC calibration.

Untreated MR was simulated on each iteration first by running the biomechanical and network model using the baseline parameters until steady-state convergence, while keeping the $MVBR^0$ at infinite. Next, we simulated the acute onset of MR by again running the compartmental model while imposing the randomly selected values of $SB{V}^{1}$, $MVBR^1$, $PV{R}^{1}$, and ${HR}^{1}$. We modeled severe MR as reported in canine experiments with a regurgitant fraction of 60±10%. For remaining steps, we employed the integrated biomechanical and network models as described in Sect. 2.4. On each simulation step, we updated the neurohormonal inputs following randomly selected data-derived time-varying curves (Bracamonte et al. 2025). SVR and HR were smoothly transitioned from their acute value to 10% above their baseline value for chronic stages of MR.

#### Simulation of pharmacological interventions

We modeled the effect of drug therapies at each level of the multiscale model. At circulatory system scale, we simulated the vasodilator effect of drugs by reducing the arterial and pulmonary vascular resistance by a factor estimated from empirical data (Table 1) (Chrysant 1998; Dell’Italia et al. 1995; Demir et al. 2016; Haynes et al. 1996; Lachance et al. 2014; Lankhaar et al. 2008; Ruzicka et al. 1993; H. Suzuki et al. 2004; S. Suzuki et al. 2012; Ting et al. 1993).

**Table 1:** Modeling the effect of pharmacological interventions

Drug/System	Hemodynamics	Organ	Molecular
bB	$SV{R}_{\mathrm{D}\mathrm{r}\mathrm{u}\mathrm{g}}^{i}=0.85 SV{R}^{i}$ $PV{R}_{\mathrm{D}\mathrm{r}\mathrm{u}\mathrm{g}}^{i}=0.95 PV{R}^{i}$	$H{R}_{Drug}^{i}=0.85 H{R}^{i}$ ${e}^{i}={e}^{0}$	${y}_{\mathrm{m}\mathrm{a}\mathrm{x}}=0$ for BAR
ARB	$SV{R}_{\mathrm{D}\mathrm{r}\mathrm{u}\mathrm{g}}^{i}=0.70 SV{R}^{i}$ $PV{R}_{\mathrm{D}\mathrm{r}\mathrm{u}\mathrm{g}}^{i}=PV{R}^{i}$		${y}_{\mathrm{m}\mathrm{a}\mathrm{x}}=0$ for AT1R
ACEi	$SV{R}_{\mathrm{D}\mathrm{r}\mathrm{u}\mathrm{g}}^{i}=0.70 SV{R}^{i}$ $PV{R}_{\mathrm{D}\mathrm{r}\mathrm{u}\mathrm{g}}^{i}=PV{R}^{i}$		${{w}_{\mathrm{A}\mathrm{n}\mathrm{g}\mathrm{I}\mathrm{I}}^{i}}_{\mathrm{D}\mathrm{r}\mathrm{u}\mathrm{g}}=0.3 {w}_{\mathrm{A}\mathrm{n}\mathrm{g}\mathrm{I}\mathrm{I}}^{i}$
CMAi	$SV{R}_{\mathrm{D}\mathrm{r}\mathrm{u}\mathrm{g}}^{i}=0.70 SV{R}^{i}$ $PV{R}_{\mathrm{D}\mathrm{r}\mathrm{u}\mathrm{g}}^{i}=PV{R}^{i}$		${{w}_{\mathrm{A}\mathrm{n}\mathrm{g}\mathrm{I}\mathrm{I}}^{i}}_{\mathrm{D}\mathrm{r}\mathrm{u}\mathrm{g}}=0.7 {w}_{\mathrm{A}\mathrm{n}\mathrm{g}\mathrm{I}\mathrm{I}}^{i}$

At organ scale, we considered the barotropic effect of beta blockers by reducing the HR to 85% of their non-treated equivalents and modeled their impact on contractility as described in Sect. 2.4.3. At molecular scale, we modeled receptor blockers or antagonists by setting the maximum activity of the targeted network node to zero $({y}_{\mathrm{m}\mathrm{a}\mathrm{x}} =0$). Some heart failure drugs such as angiotensin-converting enzyme inhibitors (ACEi) and chymase inhibitors (CMAi) do not affect cardiomyocyte function directly but affect the synthesis of AngII. We modeled the molecular effect of ACEi and CMAi by reducing the time-varying AngII stimulation by a factor of *0.3* and *0.7* respectively (Dell’Italia et al. 1995).

#### Simulation of mitral valve repair

We explored the relative role of mechanical and neurohormonal stimulation on the reversal of ventricular hypertrophy following mitral valve repair by simulating three scenarios. First, we simulated the elimination of mitral regurgitation (setting MVBR to infinite) along with reduction of neurohormonal stimulation (returning all neurohormonal inputs to baseline). Second, we simulated the elimination of mitral regurgitation while maintaining neurohormonal stimulation at chronic MR levels. Third, we reduced neurohormonal stimulation without eliminating mitral regurgitation. These three simulated interventions were introduced on steady-state chronic MR achieved after simulating six months of untreated MR, and their effect was estimated after six months of simulated recovery.

### Experimental validations

We generated a pool of 10,000 MC simulations of six months of untreated MR in dogs and calculated the 50% and 90% confidence intervals (CI50, CI90) for LVM fold change, ${V}_{\mathrm{E}\mathrm{D}}$, ${V}_{\mathrm{E}\mathrm{S}}$, EF, MAP, and MPAP at each simulated growth step. The mechanical model was calibrated using acute and baseline data (< 96 h post-MR), so we used all available biomechanical data from later stages of MR (> 96 h) for validation.

We also validated the multiscale model by comparing its predictions to data from individual studies of experimental canine MR with different drug therapy regimes (Table 2). Each of these experiments was performed on a relatively small population, typically 6–12 dogs, and the standard deviations reported for LV mass and volumes were therefore much smaller than the standard deviation of 10,000 MC simulations. To better assess how our model would predict the outcome of these individual studies, we calculated the likelihood of all 10,000 validation runs with respect to the available experimental data of the untreated MR control group of each experiment (Table 2) and selected the 500 samples with the highest likelihood relative to the experimental control data. Then, we simulated drug treatment on the 500 selected models and compared the predictions to data from the experimental treatment group. We used 500 samples because such sample size produced data variability close to the experimental variability. We complemented these validation runs by simulating the chronic effect of drugs on the original posterior PDF sample of 10,000 simulated dogs (Online Resource 3 Section ESM 3.5).

**Table 2:** Simulated drug interventions and population matching criteria

Experiment	Time between MR and treatment	Time between MR and measurements	Baseline data for population matching	Source
Early bB	0	14 and 28 days	${P}_{ED}^{0}$, $MA{P}^{0}$	(Sabri et al. 2008; Tallaj et al. 2003)
Chronic bB	0	122 days	${V}_{\mathrm{E}\mathrm{D}}^{0}$, ${V}_{\mathrm{E}\mathrm{S}}^{0}$, ${P}_{\mathrm{E}\mathrm{D}}^{0}$, ${P}_{\mathrm{E}\mathrm{S}}^{0}$,$MA{P}^{0}$	(Pat et al. 2008)
Late bB	91 days	182 days	${V}_{\mathrm{E}\mathrm{D}}^{0}$,${V}_{\mathrm{E}\mathrm{S}}^{0}$	(Tsutsui et al. 1994)
ARB	0	91 days	${V}_{\mathrm{E}\mathrm{D}}^{0}$, ${V}_{\mathrm{E}\mathrm{S}}^{0}$, ${P}_{\mathrm{E}\mathrm{D}}^{0}$, ${P}_{\mathrm{E}\mathrm{S}}^{0}$,$MA{P}^{0}$	(Perry et al. 2002)
ACEi	0	122 days	${V}_{\mathrm{E}\mathrm{D}}^{0}$, ${V}_{\mathrm{E}\mathrm{S}}^{0}$,$MPA{P}^{0}$	(Dell’italia et al. 1997)
CMAi	0	122 days	${V}_{\mathrm{E}\mathrm{D}}^{0}$, ${V}_{\mathrm{E}\mathrm{S}}^{0}$, ${P}_{\mathrm{E}\mathrm{S}}^{0}$,$MA{P}^{0}$	(Pat et al. 2010)

### Sensitivity analysis

Extensive parameter-by-parameter sensitivity analyses for the mechanical and the network models have been performed separately elsewhere (Khalilimeybodi et al. 2020; Witzenburg & Holmes 2018). In this work, we were interested in evaluating the sensitivity of predicted ventricular hypertrophy in the context of simulated mitral regurgitation on a randomly generated population. We evaluated sensitivity through two different approaches. The first approach consisted of introducing perturbations to key model inputs at different stages during simulated mitral regurgitation in a randomly generated population, and observing the change in LV mass fold change over one growth time constant (T=1140 hours). The perturbation consisted of doubling the input reaction weights of myoStrain, AngII, NE, or ET1. We introduced these perturbations at baseline, at acute MR, and chronic MR.

The second approach consisted of a z-score-normalized linear regression analysis of LV mass fold change as a function of independent model parameters ${A}^{0}$, ${B}^{0}$, ${E}^{0}$, ${V}^{0}$, SVR, PVR, SBV, $MVBR^1$, network baseline weights, ${w}_{\mathrm{b}\mathrm{a}\mathrm{c}\mathrm{k}\mathrm{g}\mathrm{r}\mathrm{o}\mathrm{u}\mathrm{n}\mathrm{d}}^{0}$, ${w}_{\mathrm{A}\mathrm{N}\mathrm{G}\mathrm{I}\mathrm{I}}^{0}$, ${w}_{\mathrm{N}\mathrm{E}}^{0}$, and ${w}_{ET1}^{0}$, and chronic network weights ${w}_{\mathrm{A}\mathrm{N}\mathrm{G}\mathrm{I}\mathrm{I}}^{\mathrm{c}\mathrm{h}\mathrm{r}\mathrm{o}\mathrm{n}\mathrm{i}\mathrm{c}}$, ${w}_{\mathrm{N}\mathrm{E}}^{\mathrm{c}\mathrm{h}\mathrm{r}\mathrm{o}\mathrm{n}\mathrm{i}\mathrm{c}}$, ${w}_{\mathrm{A}\mathrm{N}\mathrm{P}i}^{\mathrm{c}\mathrm{h}\mathrm{r}\mathrm{o}\mathrm{n}\mathrm{i}\mathrm{c}}$, ${w}_{\mathrm{B}\mathrm{N}\mathrm{P}i}^{chronic}$, and ${w}_{\mathrm{E}\mathrm{T}1}^{chronic}$ fold change. The normalized coefficients (*β*) can be interpreted as the sensitivity of the dependent variable to the parameters of the model (Sobie 2009).

All algorithms and analyses were programmed and run in MATLAB R2024a and are available along with the tabulated experimental data at https://github.com/cardiacbiomechanicsgroup/CardiacNetworkGrowth.

## Results

### Simulations of untreated MR

**Fig. 4:**
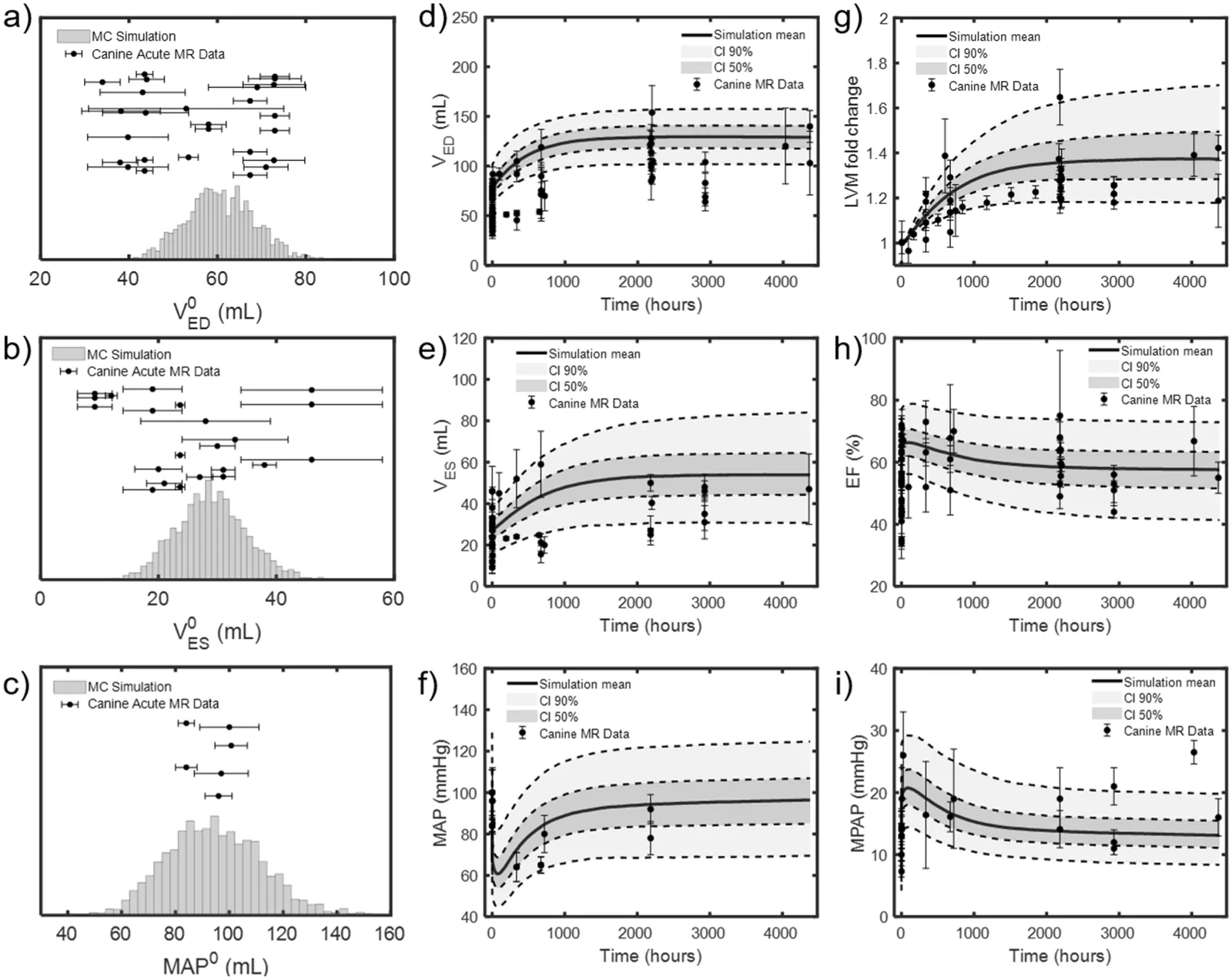
Time-varying probability distributions resulting from simulating experimental MR in N=10,000 validation runs. Baseline volumes and pressures for the simulated population (histograms) are plotted against the baseline of the experimental data reserved for validation (black markers) in panels: **a** end-diastolic volume *(V*_ED_*)*, **b** end-systolic volume *(V*_ES_), and **c** mean arterial pressure *(MAP)*. The model reproduces the evolution of key markers of ventricular growth and function in untreated MR, including **d** V_ED_, **e** V_ES_, **f** MAP, **g** LV mass fold change, **h** ejection fraction (EF), and **i** mean pulmonary arterial pressure (MPAP). MC simulations are represented by 50% confidence interval (dark shading), the 90% confidence interval (light shading), the mean (solid lines), and relevant quantiles (dashed lines). Predictions for most output variables show good agreement in both mean effect and variability with respect to experimental data (black markers) collected from multiple studies

We generated 10,000 simulations of the evolution of untreated MR over 6 months by randomly selecting parameters from continuous distributions fitted to the values retained during the MCMC calibration (Online Resource 4). Baseline LV volumes were less variable across these simulations than across the experimental studies reserved for validation, while mean arterial pressure (MAP) was more variable (Fig. 4a,b,c). We compared the ranges of model-predicted hemodynamic parameters as they evolved over six months of simulated MR to 128 experimental datasets not used for calibration, measured at a range of post-MR time points (> 96 h) in 41 studies on untreated experimental MR in dogs. Across 6 key variables, our model-predicted 90% confidence interval (CI90) contained 97 out of 128 (76%) reported experimental means, suggesting that the model captured the range of growth (Fig. 4d,g), LV function (Fig. 4e,h), and hemodynamics (Fig. 4f,i) observed over the course of experimental MR.

### Effect of pharmacological interventions during MR

**Fig. 5:**
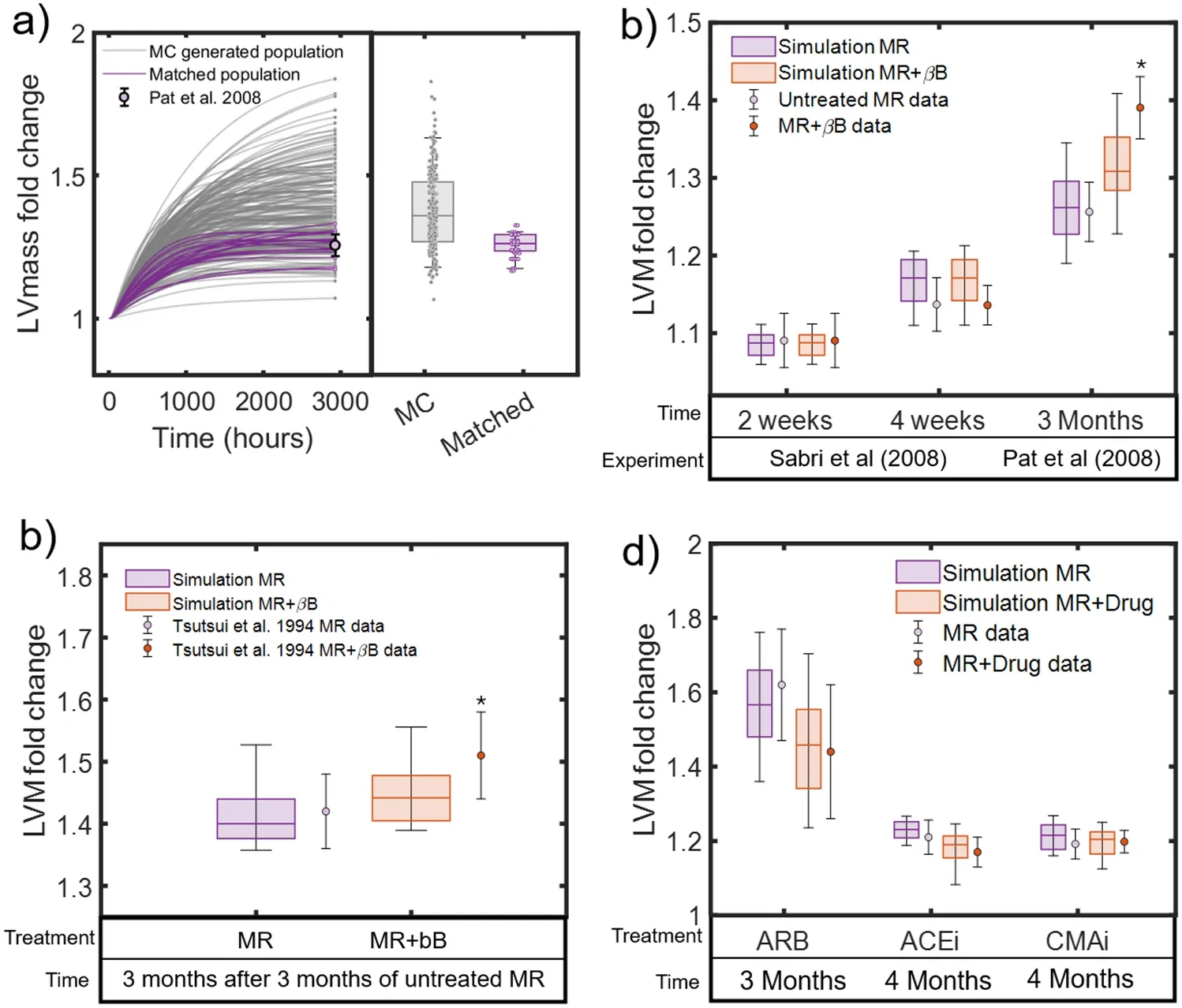
Validation of the calibrated multiscale model to data from pharmacological treatment of experimental MR. **a** Matching the characteristics and growth of the experimental untreated control group by selecting model parameters from a pre-computed population. **b** Validation of the predicted effect on LV mass of beta blocker treatment throughout the duration of experimental canine MR. **c** beta blocker (bB) treatment administered 3 months after onset of MR. **d** treatment throughout experimental MR with angiotensin receptor blockers (ARB), angiotensin-converting enzyme inhibitor (ACEi), and chymase inhibitors (CMAi)

One of the most intriguing observations in the MR literature is that beta blockers—which block a pro-hypertrophic signaling pathway in myocytes (Schäfer et al. 2001)—paradoxically exacerbate eccentric hypertrophy in long-term animal studies. Even though no bB treatment data were used for calibration, our model correctly predicted that 2–4 weeks of bB have no apparent effect on remodeling (Sabri et al. 2008), while three months of bB induce a significant increase in LV mass (Pat et al. 2008) (Fig. 5b). Our model also reproduced the observation that 3 months of bB treatment beginning after 3 months of untreated MR exacerbates LVM increases with respect to untreated controls (Fig. 5c) (Tsutsui et al. 1994). Although our model correctly predicted the existence and timing of paradoxical hypertrophy during bB treatment, it did underestimate the magnitude of the increase in LVM with long-term bB. Our model also predicted other reported effects of bB on MR hemodynamics such as the significant drop in cardiac output and ejection fraction after the administration of bB reported by Nagatsu et al. 1994a, b and Sabri et al. 2008 (results not shown).

**Fig. 6:**
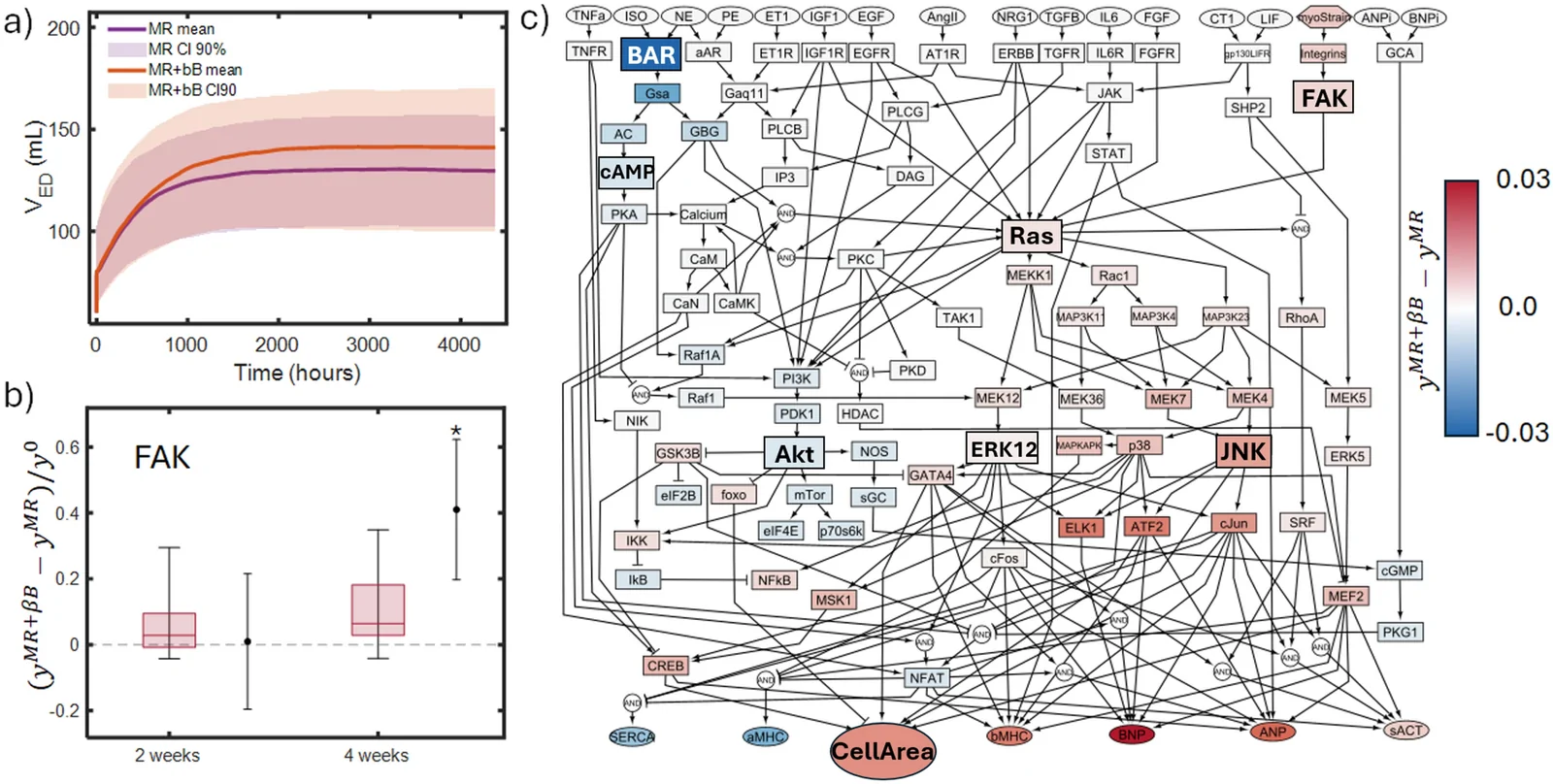
Chronic effect of beta blockers on **a** end-diastolic volume, **b** focal adhesion kinase activity fold change according to our model (red box plot) and experimental data by Sabri et al (2008), and **c** activity of network models. Beta blockade zeroes the activity of the beta adrenergic receptor node (BAR at the top left of the network), depressing calcium signaling pathways, while the dilation of the ventricle increases mechanical stimulation (myoStrain at the top right of the network) and the activation of mechanotransduction pathways. Nodes key to discussion are highlighted with larger boxes and font

One advantage of mechanistic models is the ability to interrogate them to better understand complex interactions that might underpin a particular response. In our calibrated model, blockade of the beta adrenergic receptor (BAR) depresses cAMP-mediated signaling pathways and ultimately produces deactivation of Akt, which acts to reduce hypertrophy through Akt-FoxO signaling (Skurk et al. 2005). However, this effect is outweighed by the fact that bB also reduces LV myocardial contractility, shifting the LV toward higher volumes at later time points (Fig. 6a). Increased end-diastolic stretch increases mechanical stimulation (myoStrain) in the signaling network, which enhances Integrin engagement, the phosphorylation of focal adhesion kinase (an effect reported by Sabri et al. 2008) and activates pro-hypertrophic pathways mediated by Ras, such as MAPK-JNK and ERK12-GATA4 signaling (Kehat and Molkentin 2010; Muslin 2008)(Fig. 6b,c).

Other drugs that affect known hypertrophic signaling pathways have also been tested during experimental MR in dogs and found to have no significant effect on LV mass compared to untreated controls (Dell’italia et al. 1997; Pat et al. 2010; Perry et al. 2002). For completeness, we simulated treatment with ARB, ACEi, and chymase inhibitors and compared the model-predicted effects with published data. Overall, both the magnitude and variability of the model-predicted drug effects agreed well with published reports (Fig. 5d). Finally, we evaluated the effect of drugs on the general simulated population of 10,000 animals, which resulted in the same trends as observed in Fig. 5 (bB exacerbates hypertrophy, ARB, ACEi, and CMAi tend to prevent hypertrophy, with ARB producing the largest effect), but in most cases higher variability, consistent with the high variability of the full simulated population (Online Resource 3 Section ESM 3.5).

### Simulations of surgical correction of mitral valve regurgitation.

In contrast to the wealth of published hemodynamic and neurohormonal data following creation of experimental regurgitation, we identified only a handful of studies of reverse remodeling following correction of experimental MR, and none of those studies measured changes in neurohormonal activity. We were therefore unable to directly validate model-predicted remodeling following correction of MR against published studies. Instead, we used the calibrated model to explore the relative role of mechanical and neurohormonal stimuli on predicted reversal of hypertrophy following the elimination of mitral regurgitation, and then compared multiple model scenarios to the limited available data.

**Fig. 7:**
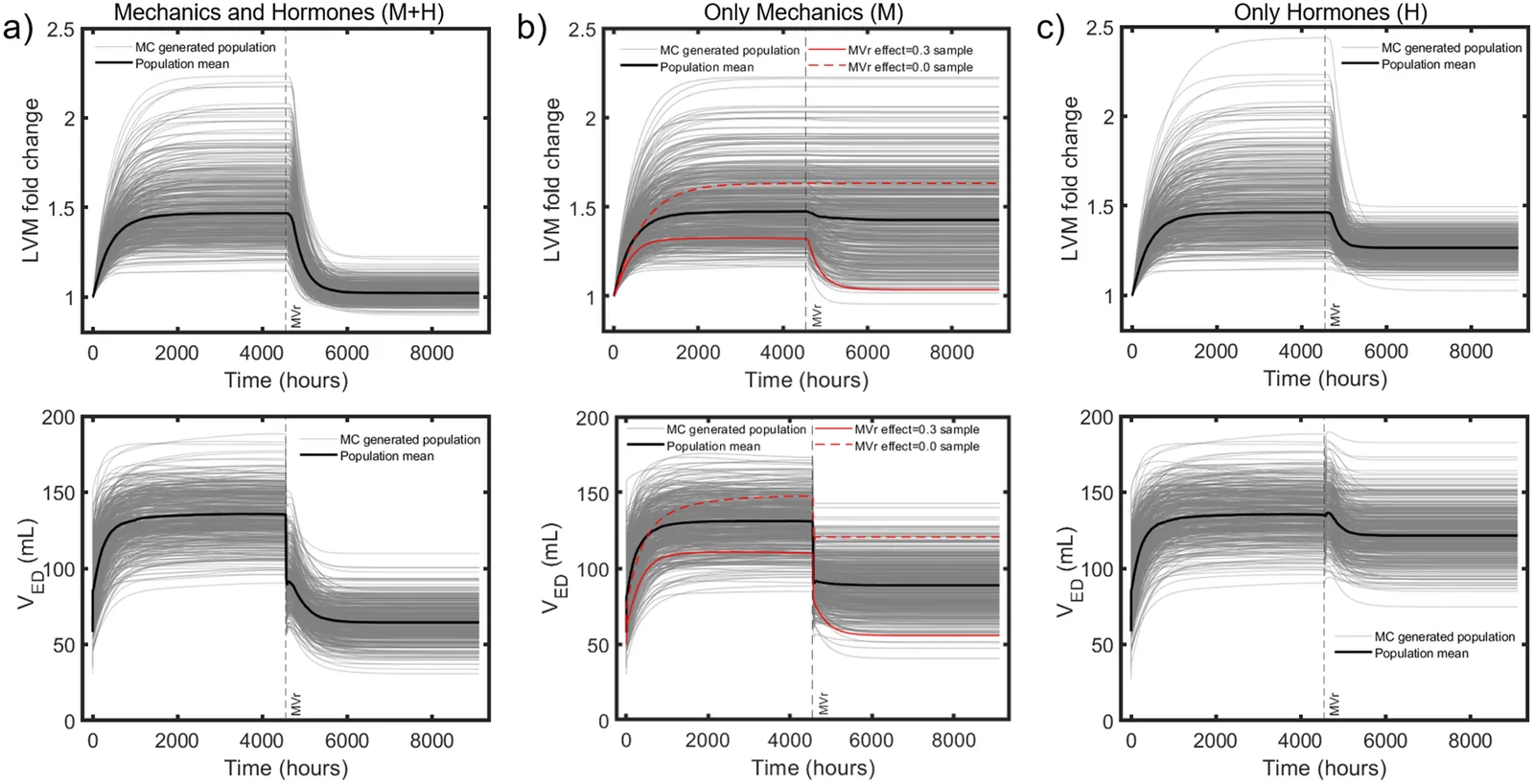
Simulating the reversal of ventricular hypertrophy following the elimination of mitral regurgitation (MVr). We considered three cases: **a** eliminating mitral regurgitation and restoring baseline neurohormonal stimulation (M+H), **b** eliminating mitral regurgitation but sustaining elevated neurohormonal stimulation (M), and **c** restoring baseline neurohormonal stimuli but sustaining volume overload (H). The simulated LVM response to eliminating mechanical volume overload (panel b) was variable, from cases that reversed most of LV growth (solid red line), to cases that remained unaffected (dashed line), resulting in a small mean effect. MVr effect is defined as the difference between preoperative to chronic postoperative LVM fold change

We simulated three different scenarios: the case in which mitral valve repair eliminates regurgitation and returns neurohormonal stimuli to baseline levels; the case in which mitral repair eliminates regurgitation but has no impact on neurohormonal activity; and an artificial comparison case where neurohormonal activation returns to baseline despite continuing MR. We found that eliminating MR and normalizing all neurohormonal input levels led to near-complete reversal of both hypertrophy and dilation (Fig. 7a). We found that the mechanical elimination of volume overload explained most of the predicted reduction in ${V}_{ED}$ (Fig. 7b). We noted some cases in which the elimination of the regurgitant flow alone produced essentially no reduction in LVM despite a substantial drop in ${V}_{ED}$ (Fig. 7b). These unresponsive cases constitute almost 38% of simulations when neurohormonal activity is sustained elevated after repair and corresponded to simulations with the greatest neurohormonal activity and hypertrophy (LVM fold change >1.5) prior to repair. Eliminating the neurohormonal activity without resolving the regurgitant flow revealed that changes in hormonal stimulation accounted for most of the predicted reduction of LVM following simulated repair, while holding smaller influence on ${V}_{ED}$ (Fig. 7c).

**Fig. 8:**
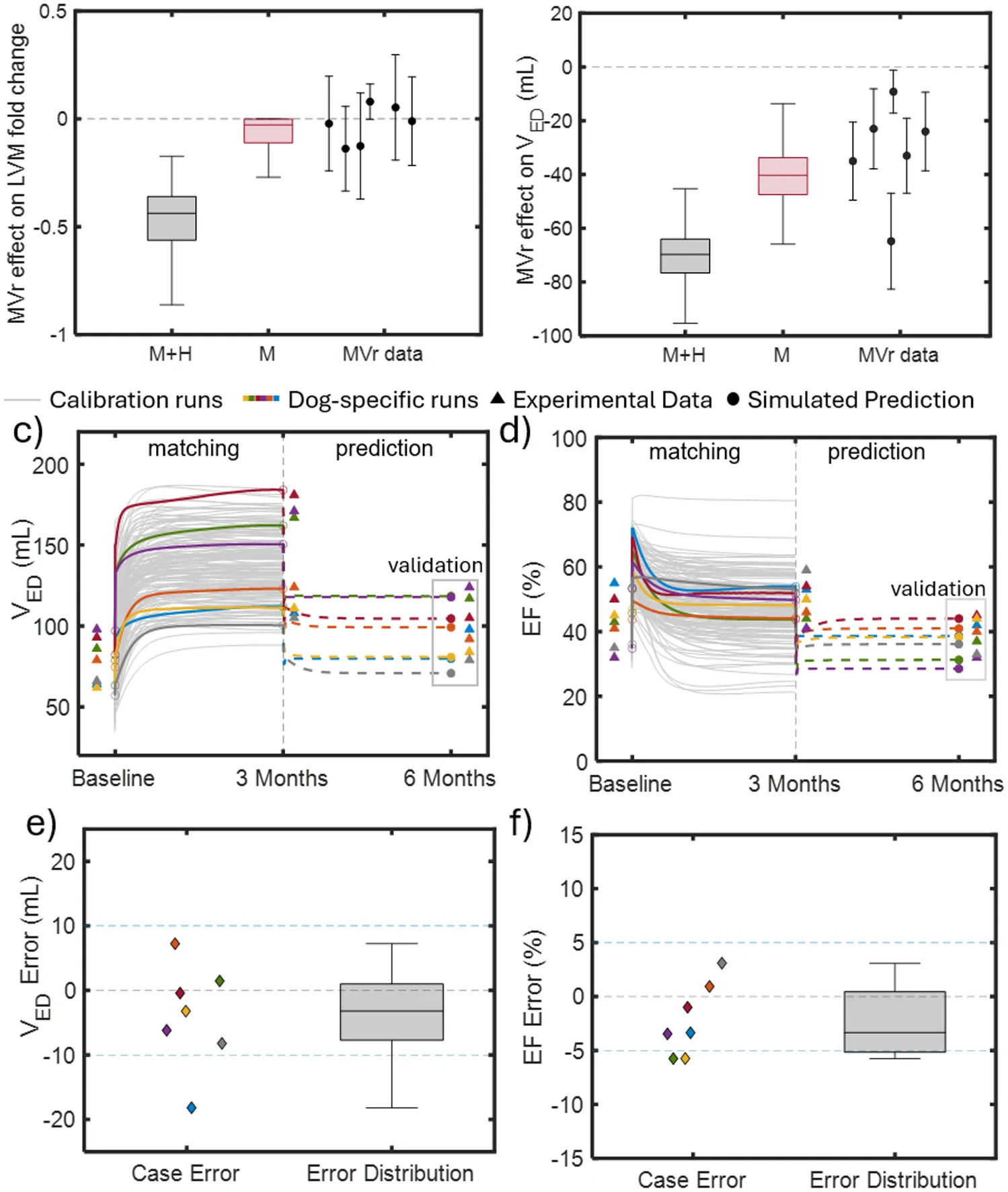
Comparing simulated predictions of the effect of mitral valve repair (MVr) on **a** *LVM* and **b** end-diastolic volume *(V*_ED_) and compared to data from multiple studies on experimental mitral valve replacement in dogs. We simulated the response to MVr by eliminating the regurgitant flow and restoring normal neurohormonal stimuli (M+H), and by eliminating regurgitant flow but sustaining elevated neurohormonal stimuli (M). Experimental data is more consistent with by M case simulations. We explored the matching of M case simulations to individual dog pre-operatory **c** *V*_ED_ and **d** *EF* data from the mitral replacement experiments by Ishihara et al. (1992). The matched simulation runs were used to generate individualized predictions. We calculated the error between individual predictions and the corresponding experimental measurements 3 months after valve replacement, with **e** *V*_ED_ and **f** ejection fraction

Next, we compared our model scenarios to the results of five longitudinal studies of mitral regurgitation and correction (Ishihara et al. 1992; Nagatsu et al. 1994a, b; Nakano et al. 1991; Spinale et al. 1993; Zile et al. 1993). In these experiments, MR was left untreated for three months, after which the damaged valves were replaced with a xenograft and the dogs left to recover for three more months. All these studies found that valve replacement had little effect on LVM despite a significant drop in ${V}_{ED}$, and thus are more consistent with our simulations that assume persistent neurohormonal stimulation after the elimination of mitral regurgitation (Fig. 8a,b).

We also explored the ability of our calibrated multiscale model of canine MR to reproduce long-term outcomes following mitral valve replacement in individual dogs as reported by Ishihara et al 1992 (Ishihara et al. 1992). ${V}_{\mathrm{E}\mathrm{D}}$, EF (triangles, Fig. 8c,d), pulmonary pressure, and end-systolic stress were measured or calculated at baseline (before MR), before valve replacement, and at the 6-month study endpoint. From the pool of 10,000 simulations of untreated MR, we identified individual runs (solid color lines) that best matched baseline and chronic MR measurements for the given regurgitant fraction using a statistical likelihood criterion. Then, we simulated valve replacement by eliminating the regurgitant flow (dashed color lines) while maintaining elevated neurohormonal activity. Finally, we evaluated the simulation error against experimental data. Although our model was only calibrated against data on pre-repair remodeling, the matching procedure employed here predicted 3-month post-repair EF with an average error of just 3% and ${V}_{\mathrm{E}\mathrm{D}}$ with a mean error of just 4 mL (Fig. 8e,f).

### Sensitivity analysis

#### Multiscale model sensitivity to parameter perturbations

**Fig. 9:**
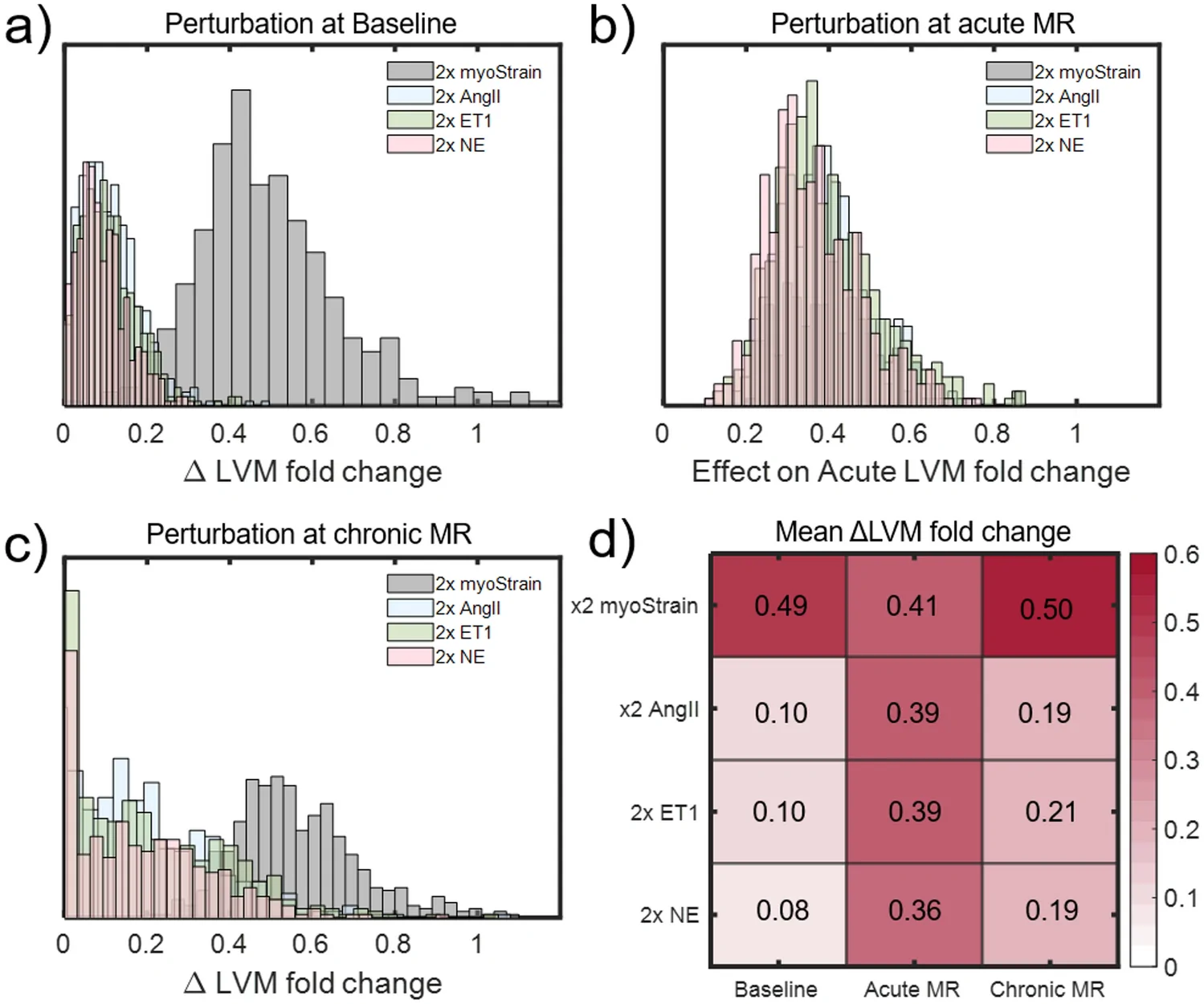
Perturbation-based sensitivity analysis consisting of measuring the effect of doubling the weight of relevant network inputs on *LVM* fold change over 1140 h at **a** baseline, **b** acute MR, and **c** chronic MR. **d** Heatmap of mean change on *LVM* fold change following perturbations on network inputs

Doubling any neurohormonal input at baseline yielded a similar response on LVM growth over one time constant (T=1140 hours), with an approximate mean growth of 10%. In contrast, doubling the myoStrain input yielded a significantly higher response, with a 49% LV mass growth within one time constant *T* (Fig. 9a). This is a direct consequence of the design of the strain–myoStrain mapping function (Sect. 2.4.1), which maximizes the sensitivity of the hypertrophic response to acute levels of fiber strain. When the perturbation is introduced in acute MR, all stimuli (neurohormonal and mechanical) yielded a similar response on LV mass growth with a mean effect close to 40%. This suggests that doubling the acute activity of any of the stimuli considered here saturated the growth response (Fig. 9b).

When the perturbation is introduced in chronic stages of MR, we observed a similar response to doubling any of the neurohormonal inputs, with a mean hypertrophic effect of 20%, with a distribution skewed to the left (Fig. 9c). The significant number of simulations where doubling neurohormonal inputs leads to no hypertrophic response suggests that, in many cases, those pathways were already saturated before the perturbation. Notably, the response to doubling myoStrain stimulation in the chronic stages of MR was significantly larger than to any of the perturbations of neurohormonal inputs, and close to the response to myoStrain perturbation at baseline (Fig. 9d). This result suggests the multiscale model is as sensitive to mechanical stimulation in chronic stages of MR as it is at a healthy baseline. This results from the fact that, unlike neurohormonal stimuli that remain chronically elevated in late stages of MR, the ventricular growth compensates mechanical strain, which resets the mechanotransduction pathways back to their baseline.

#### Normalized linear regression analysis

The normalized linear regression analysis suggests that mechanical parameters play a marginal role in LV mass growth compared to the influence of other parameters in the network model. We found the weight of the background reactions $({\beta}_{w \mathrm{b}\mathrm{a}\mathrm{c}\mathrm{k}\mathrm{g}\mathrm{r}\mathrm{o}\mathrm{u}\mathrm{n}\mathrm{d}}=-0.62)$ showed an inverse proportion to LV mass growth. This owes to the fact that a higher background activity of the network increases the baseline CellArea, thereby narrowing the range of possible growth and reducing the overall network’s sensitivity to hypertrophic stimuli. Chronic activity of ET1 appeared to be the most influential pro-hypertrophic stimulus $\left({\beta}_{wET{1}^{\mathrm{c}\mathrm{h}\mathrm{r}\mathrm{o}\mathrm{n}\mathrm{i}\mathrm{c}}}=0.57\right)$, followed by chronic stimulation by AngII and NE (Online Resource 5). Furthermore, we observed that the reversal of ventricular hypertrophy following elimination of mitral regurgitation while maintaining elevated neurohormonal activity displayed a parameter sensitivity map that mirrored that of ventricular growth, with elevated neurohormonal activity inversely related to the reversal of cardiac hypertrophy (Online Resource 5).

## Discussion

In this work, we developed a multiscale model of ventricular hypertrophy consisting of components representing ventricular mechanics, systemic hemodynamics, and cardiomyocyte molecular signaling, and calibrated it using a machine learning algorithm and data from 76 animal experiments. The resulting Bayesian model generates probabilistic predictions of the hypertrophic response to experimental MR in dogs, and how it is modulated by drug therapies that matched independent experimental data. Model-predicted 90% confidence intervals contained 76% of 128 experimental measurements of LV size, function, and systemic hemodynamics from a variety of independent experimental studies of untreated MR not used in its calibration. LV volumes accounted for most of the experimental data falling outside the model-predicted 90% confidence interval in simulations of untreated MR (Fig. 4d and e). This may reflect the fact that most studies reporting acute data—and therefore used for calibration—were performed in larger dogs with baseline ${V}_{ED}$ values well above 60 mL (Fig. 2c), while many of the validation studies employed smaller dogs with baseline ${V}_{ED}$ values below 60 mL (Fig. 4a). Even though the model was only calibrated using data on untreated experimental MR, the model-predicted CI90 for simulations of experimental MR plus drug treatment encompassed all published experimental means. These experimental validations demonstrate the capabilities of our multiscale model and calibration approach to generate time-resolved predictions not only of mean effects, but also of experimental variability across a population.

Notably, reproducing the effects of bB required a multiscale approach. Multiple studies have demonstrated that bB protects against cardiomyocyte hypertrophy induced by agonist exposure in vitro and in vivo (Boluyt et al. 1995; Fung et al. 2003; Grimm et al. 1999; Hanada et al. 2008; Kitagawa et al. 2004; Schäfer et al. 2001; Wang et al. 2020). However, bB paradoxically exacerbates hypertrophy in experimental volume overload in dogs and rats (Pat et al. 2008; Seqqat et al. 2012). Previously, we found the network model of cardiomyocyte signaling employed here was sufficient to reproduce the protective effect of bB against the infusion of agonists, but not its paradoxical influence on hypertrophy during experimental MR (Bracamonte et al. 2025). By contrast, this multiscale model incorporating the same signaling network produced paradoxical worsening of hypertrophy by long-term bB treatment through interactions among the various model components. In the multiscale model, bB directly curbed myocyte hypertrophy by inhibiting the cascade of signaling reactions downstream of the beta adrenergic receptor (BAR), but this was outweighed by the fact that bB also reduced LV contractility, leading to higher diastolic volumes and myocyte stretching and thereby promoting hypertrophy through mechanotransduction pathways downstream of the myoStrain and Integrin nodes (Fig. 6). Our model-driven hypothesis that bB activates myocyte mechanotransduction in VO is further supported by reports of increased FAK activity in dogs and rats that received bB when compared to untreated VO controls (Sabri et al. 2008; Seqqat et al. 2012).

When exploring predicted responses to elimination of mitral regurgitation, our model suggests that a full reversal of ventricular hypertrophy (mass) and dilation (volumes) requires both the elimination of volume overload and the restoration of the baseline neurohormonal state. This is consistent with the reported regression of ventricular hypertrophy and restoration of normal plasma concentration of natriuretic peptides, angiotensin, and renin activity following the closure of aortocaval fistulas in rats (Abassi et al. 2001; Gerdes et al. 1995). In our model, eliminating regurgitation is essential for restoring normal or near-normal volumes, while normalizing the hormonal environment is more important for regression of LV mass. In some simulations where neurohormonal stimulation was particularly strong during MR and remained elevated following elimination of mitral regurgitation, the model predicted no regression of mass despite a significant drop in end-diastolic volume (Fig. 7b). Understanding which factors may limit reverse remodeling may be of practical relevance given that clinical studies have shown that mitral valve repair produces a significant long-term reduction in LVM in some, but not all, asymptomatic patients (Hibino et al. 2023; Le Tourneau et al. 2019; Quintana et al. 2014; Zheng et al. 2023, 2024), and produces symptomatic improvement but no reversal of hypertrophy in patients with heart failure (Joung et al. 2021; Marciniak et al. 2011; Zhou et al. 2022). Taken together, these reports and our models suggest that measurement of neurohormonal activation before and after mitral repair in individual patients might provide additional insight into their likely postoperative outcomes.

Cardiac hypertrophy is known to result from a combination of genetic, mechanical, and neurohormonal factors (Frank et al. 2018; Sahli Costabal et al. 2019a, b; Toischer et al. 2010; You et al. 2017). Yet many groups—including ours—have published successful computational models of hypertrophy driven by changes in mechanics alone; some examples are reported by Kerckhoffs et al. 2012; Oomen et al. 2022; Peirlinck et al. 2019; Sharifi et al. 2021; Witzenburg & Holmes 2017, 2018; Yoshida & Holmes 2021. We speculate that in situations where hypertrophy is triggered by an experimental intervention (such as aortic banding or creation of an arteriovenous fistula) that produces large changes in both mechanics and the neurohormonal environment, mechanics can serve as a surrogate for the many hypertrophic stimuli that are changing at once. This notion is supported by the observation that, in acute stages of MR, the perturbation of any one of the already exacerbated stimuli (neurohormonal or mechanical) yielded a similar effect on growth (Fig. 9b), making their contributions to rapid hypertrophy practically indistinguishable. However, mechanics-only approaches have had more difficulty reproducing regression of hypertrophy following correction of a hemodynamic overload (Oomen et al. 2022; Sharifi et al. 2021; Yoshida et al. 2020) and clearly break down where there is a need to account for drug treatments alone or in combination. An interesting feature of the proposed multiscale model is that neurohormonal stimuli remained elevated in chronic stages of MR while the mechanical stimulation was fully compensated by growth (Fig. 9c), restoring differential sensitivity to mechanical vs. further neurohormonal stimulation. The network model of hypertrophic signaling employed here provides critical integration across pathways engaged by multiple hypertrophic stimuli in order to predict responses to drugs in the setting of altered mechanics or to treatments that impact both mechanics and circulating neurohormonal factors (Estrada et al. 2021; Sharifi et al. 2021; Yoshida et al. 2022). We recently showed that the network model alone can reproduce many single-drug, multidrug, and overload-plus-drug responses in animals in situations where LV mechanics are well characterized (Bracamonte et al. 2025). Thus, the full multiscale approach presented here may only be necessary when considering interventions such as bB treatment or repair of MR that clearly alter both mechanics and hypertrophic signaling.

The mechanistic multiscale model presented here offers many advantages, including the ability to predict responses to single or multiple interventions for which it was not specifically calibrated. However, one clear disadvantage is the large number of model parameters that require calibration with respect to other models of cardiovascular therapy (Bracamonte & Holmes 2025). We found no published study that provided sufficient information to fully calibrate the model presented here. We therefore turned to a Bayesian approach that allowed us to integrate data from multiple experiments and animal models and propagate the experimental uncertainty into the outputs while using the structure of the multiscale model to account for interdependence among parameters. The enhancement of multiscale models with Bayesian approaches takes advantage of the wealth of data from previous studies, and reduces the amount of data required for model calibration when compared to black-box machine learning models and conventional statistical methods (Buoso et al. 2021; Kissas et al. 2020; Sahli Costabal et al. 2020; Sahli Costabal et al. 2019a, b). The resulting model generates predictions in the form of probability distributions of expected responses to simulated interventions. Thus, this model has the advantage of providing information about both the expected size effect and the expected variability. This information may be useful to identify potential therapeutic targets and guide the experimental design of clinical and in vivo testing.

We also explored using a pre-generated pool of simulations to identify the subset of parameters that most likely describe a given subpopulation and then predict the effect of a given intervention in that specific subpopulation. Beyond comparing model performance to individual studies in which baseline characteristics differ, this approach might be useful in identifying subgroups of patients that are particularly sensitive to a prospective treatment or may develop adverse side effects. While the use of statistical likelihood to select the subset of parameters enables the matching of the known patient group data, the adequate size of the parameter subset will depend on the data uncertainty and the pool of simulations.

Recognizing that there will never be enough data from a specific patient to fit the number of model parameters required for our simulations (Bracamonte & Holmes 2025), we also explored whether selecting a single case from a pre-generated pool that best matches the pre-treatment state for a specific patient could allow for individualized predictions of the response to an intervention. This method produced promising results when applied to a single study of experimental MR followed by repair in dogs, even though matching was based only on pre-treatment ${V}_{\mathrm{E}\mathrm{D}}$ and EF. Given that neurohormonal factors drive most of the hypertrophy and potential regression in our model, we suspect that measurement of serum levels of relevant hypertrophic agonists would further enhance our ability to make individual predictions by matching pre-intervention characteristics.

## Limitations

While the generation of predictions as probability distributions is one of the most attractive features of our Bayesian approach, some of the choices we made when implementing it may introduce unnecessary variability. First, we treated the levels of circulating hormones as independent of each other and of MR severity. However, experiments and clinical data suggest that circulating hormone concentrations are correlated with the severity of the cardiac insult. Incorporating such correlations into random sampling might improve predictions of the likely biological variability in the responses modeled here. The integration of data from multiple sources and animal models is also a source of uncertainty. Factors such as differences in the severity of volume overload, animal size, species, and sex are reflected in variability in the calibrated model’s predictions.

Although useful for this exploratory work, our model of adrenergic compensation of contractility is simplistic—representing only complete compensation or no compensation—and does not account for variations in circulating NE levels or receptor availability and sensitivity, which can change during eccentric remodeling and the transition to heart failure. Similarly, vasoconstriction/vasodilation responses to MR and drug therapies are currently prescribed ad hoc based on empirical data. Furthermore, hemodynamic effects of drugs were specified using a fixed percentage of baseline values, rather than through the Bayesian approach employed for other parameters. Because the sensitivity analysis (Online Resource 5) indicated low sensitivity of growth to HR and SVR, we expect the impact of these simplifications on predicting growth to be small. Adding a reflex model that dynamically couples blood pressure, blood flow, vascular resistance, and neurohormonal responses could avoid many of the ad hoc choices employed here while accounting for the coupling of hormone levels to one another and to the severity of dysfunction mentioned in the previous paragraph. The fact that reflex compensation to preserve mean arterial pressure (MAP) is absent from our models may explain why the range of MAP in our calibrated model is so much larger than the range reported in studies used for validation (Fig. 4c).

The biomechanical system simulated in this work does not include a model for the left atrium. In the circuit employed here, the pulmonary venous capacitance provides much of the passive buffering of (forward) pulmonary venous flow and (backward) regurgitant flow that an atrial compartment would normally provide, enabling the model to reproduce the experimental LV end-diastolic pressure. However, the system cannot account for active atrial emptying or remodeling-related effects, such as atrial chamber dilation and changes in muscle stiffness. Calibrating atrioventricular dynamics requires continuous pressure–volume or pressure–flow curves that are not provided in most published experimental studies of MR. In future studies, we plan to expand this model to represent atrioventricular mechanics and transvalvular hemodynamics by incorporating an additional contractile chamber for the LA and acquiring the data needed to properly calibrate it. We also reduced the number of parameters to be calibrated by assuming that the properties of the right ventricle are proportional to the properties of the left ventricle and neglecting any RV-specific remodeling or changes in function during mitral regurgitation. In reality, the regurgitant flow can eventually lead to pulmonary congestion and challenges to blood oxidation that may affect the mechanics of the right heart, so more attention should be paid in future modeling to the evolution of the right heart structure and function in MR.

For this work, we evaluated the model’s capacity to simulate ventricular growth history in untreated and treated MR by counting the number of experimental means falling within the predicted confidence intervals. While this straightforward method provides an intuitive measure of the simulation’s agreement with experimental data, it is not a rigorous goodness-of-fit metric. A goodness-of-fit test that properly accounts for all sources of uncertainty would be necessary for a rigorous comparison between the proposed model and other methods in future work. For the implementation of the Metropolis–Hastings algorithm, we assumed a symmetric proposal function, in which the selection of the new parameter set is random and independent of the current parameter set. While this method is easy to implement and works well for parameter spaces where all parameters are independent, it yielded a relatively low acceptance rate (around 5%) during calibration of the biomechanical model. The physiology-driven covariance between model parameters A and B and SVR and HR may be better managed with a larger acceptance rate via a more sophisticated MCMC algorithm, such as Hamiltonian Monte Carlo (Andrieu et al. 2003; Radivojević and Akhmatskaya 2019).

Our computational model and calibration algorithm integrate animal data on volume overload. To apply these methods to human clinical data, important differences between natural and experimental mitral regurgitation must be addressed. In dog models, mitral regurgitation is induced by cutting the chordae tendineae, causing acute, severe regurgitation in an otherwise healthy heart, unlike the gradual onset of naturally occurring MR. Nevertheless, a number of key pathways and mechanisms underlying MR-induced growth and remodeling appear to be activated in both animals and patients (Carabello 1996; Dillon et al. 2012). Thus, we believe that the basic structure of our multiscale model will provide a useful starting point for modeling human MR, even if considerable recalibration is required.

## Conclusions

We assembled a multiscale model of ventricular hypertrophy coupling ventricular mechanics, systemic hemodynamics, and cardiomyocyte molecular signaling. To calibrate a total of 11 mechanical and 4 molecular unknown parameters, we employed Bayesian statistics in the form of a machine learning algorithm to assimilate data from 76 animal experiments. The calibrated model not only predicted the history of hemodynamic and ventricular growth markers during untreated experimental MR in dogs, but it also reproduced the modulating effect of drug therapies on VO-induced cardiac hypertrophy. Notably, this multiscale model reproduced the exacerbation of hypertrophy by chronic beta blockade treatment in the context of experimental VO, an effect that an isolated cardiomyocyte network model could not capture. The model suggests that the suppression of contractile compensation by beta blockers induces cardiac dilation, and with it, the activation of hypertrophic mechanotransduction pathways that outweighs the direct interruption of beta adrenergic signaling. This interesting result highlights how the effects of drug therapies at different scales may interact and lead to unexpected outcomes, making a clear case for the use of multiscale modeling in the study of biological and biomedical systems.

When simulating the elimination of volume overload, as a model of mitral valve repair, our multiscale model suggests that both the elimination of the mechanical overload and the normalization of neurohormonal stimuli are necessary for a full reversal of cardiac hypertrophy. Furthermore, we found that sustaining elevated neurohormonal stimulation in some simulated cases may prevent the reversal of cardiac hypertrophy even if the mechanical overload is eliminated. The model, calibrated using data from multiple experiments performed in different animal samples with a Bayesian technique, generates probabilistic predictions with ranges that represent the variability of a broad population, with potential applications in designing large population studies and clinical trials. We further explored the advantages of this probabilistic feature by identifying subsets of model parameters that best represent the characteristics of a subpopulation. These subsets were selected from a pre-generated pool of simulations and then used to predict the effect of interventions on subpopulations of known characteristics. This selection method may be a convenient tool for identifying and studying subpopulations of interest and developing tools for precision medicine.

## Supplementary Information

Below is the link to the electronic supplementary material.

Supplementary file1 (XLSX 39 kb)

Supplementary file2 (DOCX 971 kb)

Supplementary file3 (DOCX 1626 kb)

Supplementary file4 (TIF 552 kb)

Supplementary file5 (TIF 364 kb)

## Data Availability

Tabulated data, source code for the multiscale model, and the outputs of the simulation runs used for validation are publicly available via GitHub links referenced in the body of the manuscript. Tabulated data, source code for the multiscale model, and outputs are publicly available via GitHub repository https://github.com/cardiacbiomechanicsgroup/CardiacNetworkGrowth
