# Supplementary material for: Multiscale model calibrated with a Bayesian algorithm predicts long-term outcomes of treatments for mitral valve regurgitation in animal models
Source: Biomech Model Mechanobiol. 2026 Sep 20;25(5):107. doi: 10.1007/s10237-026-02123-0 (PMC13590452; doi:10.1007/s10237-026-02123-0)
Supplement: Supplementary file 1 [file 10237_2026_2123_MOESM2_ESM.docx]

**Multiscale model calibrated with a Bayesian algorithm predicts long-term outcomes of treatments for mitral valve regurgitation in animal models**

Johane H. Bracamonte, Lionel Watkins, Jeffrey J. Saucerman, Jeffrey W. Holmes

**Supplementary Material ESM2. Tabulated Data Sources.**

**ESM2.1. Sources of experimental data for calibration of mechanical model.**

| **Experimental measurement** | **Sources** |
| --- | --- |
| **Healthy/base end diastolic myocardial fiber stretch** | (Fomovsky et al., 2012; Ross et al., 1971; Ross & McCullagh, 1972; Spotnitz et al., 1966) |
| $V_{ED}$, $V_{ES}$, $P_{ED}$, $P_{ES}$, $dP/dt_{max}$, $MAP$, $DBP$, $MPAP$, $EF$, $rF$ | (Berko et al., 1987; Carabello et al., 1989a; Imamura et al., 1994; Katayama et al., 1988; Kleaveland et al., 1988; Nakano et al., 1991; Prasad et al., 1977; Tsutsui et al., 1994) |

# ESM2.2. Sources of experimental data from canine MVR and experimental VO in rats used to fit the network model of cardiomyocyte hypertrophy.

| **Experimental measurement** | **Sources** | |
| --- | --- | --- |
|  | **Canine MVR** | **VO in rats** |
| **Cell growth** | | |
| **Myocyte Cell Area/Length** | (Dell’italia et al., 1997; Kihara et al., 1988; Pat et al., 2008; Perry et al., 2002; Spinale et al., 1993; Tsutsui et al., 1994; Urabe et al., 1992) | (Du et al., 2010; Jobe et al., 2009) |
| **Concentration in plasma of hypertrophy-related hormones and enzymes** | | |
| **ANP** | (Asano et al., 1999, 2001; Hori et al., 2010; Ichiki et al., 2013) | (Abassi et al., 2011; Huang et al., 1992; Langenickel et al., 2000; Pagel et al., 2002; Willenbrock et al., 1997) |
| **BNP** | (Asano et al., 1999, 2001; Hori et al., 2010; Ichiki et al., 2013) | (Langenickel et al., 2000; Wang et al., 2012) |
| **ANGII** | (Ichiki et al., 2013; Perry et al., 2002; Tallaj et al., 2003) | (Bauer et al., 1997; Ishiye et al., 1995; Pagel et al., 2002; Shuji et al., 2000; Suzuki et al., 2004; Wang et al., 2012) |
| **NE** | (Hankes et al., 2006; Lee et al., 1985; Nagatsu et al., 1994; Tsutsui et al., 1994) | (Huang et al., 1992; Kristen et al., 2002, 2006; Oka et al., 1993; Suzuki et al., 2004; Willenbrock et al., 1997) |
| **ET1** | (Cavero et al., 1990; Ray et al., 2008) | (Gardner et al., 2010; Ishiye et al., 1995) |
| **Intracellular signaling protein activity/phosphorylation** | | |
| **FAK** | (Pat et al., 2010; Sabri et al., 2008) | (Seqqat et al., 2012) |
| **Akt** | (Sabri et al., 2008) | (Dent et al., 2007) |
| **ERK12** | (Sabri et al., 2008) | (Kolpakov et al., 2009; Zhang et al., 2010) |
| **ERK5** | (Liu et al., 2013) | -- |
| **JNK** | (Sabri et al., 2008) | (Kolpakov et al., 2009) |
| **p38** | (Liu et al., 2013; Sabri et al., 2008) | (Kolpakov et al., 2009) |
| **STAT** | -- | (Kolpakov et al., 2009) |
| **cGMP** | (Liu et al., 2013; Trappanese et al., 2015) | (Arnal et al., 1993; Pagel et al., 2002) |
| **ELK** | -- | (Zhang et al., 2010) |
| **Protein abundance** | | |
| **ANP** |  | (Fareh et al., 1996; Lachance et al., 2014; Langenickel et al., 2000; Yamakawa et al., 2000) |
| **BNP** | (Zheng et al., 2009) | (Fareh et al., 1996; Langenickel et al., 2000; Yamakawa et al., 2000) |
| **αMHC** | (Imamura et al., 1994; Matsuo et al., 1998) | (Freire et al., 2007; Lachance et al., 2014; Pu et al., 2009). |
| **βMHC** |  |  |
| **SERCA2** | (Zheng et al., 2009) | (Pu et al., 2013; Wojciechowski et al., 2010) |

# ESM2.3. Time-varying curves of neurohormonal alterations for network inputs in volume overload.

| Species | **Function** | **a** | **b** | **C** |
| --- | --- | --- | --- | --- |
| ANP | $\frac{w_{ANP}}{{w_{ANP}}^{0}}=at/\tau+b$ | $1.0747\pm0.5612$ | $3.8311\pm2.0542$ | -- |
| BNP | $\frac{w_{BNP}}{{w_{BNP}}^{0}}=at/\tau+b$ | $1.5746\pm0.2508$ | $1.05\pm0.05$ | -- |
| ET1 | $\frac{w_{ET1}}{{w_{ET1}}^{0}}=a$ | $3.0\pm0.98$ | -- | -- |
| NE | $\frac{w_{NE}}{{w_{NE}}^{0}}=at/\tau+b$ | $0.1213\pm0.0802$ | $1.4610\pm0.211$ | -- |
| AngII | $\frac{w_{AngII}}{{w_{AngII}}^{0}}={ae}^{-t/b}+c$ | $4.46 \pm0.98$ | $0.533 \pm0.178$ | $2.757 \pm0.793$ |

**Figure ESM2.1.** Graphical representation of time-varying curves of network inputs during mitral regurgitation. ANGII, NE, ANPi, BNPi, and ET1 curves are derived from data from experiments of volume overload in rats and mitral regurgitation in dogs. Time varying curves of strain are either generated from simple geometrical assumptions (yellow lines) or obtained through the evaluation of the multiscale model (gray). The derivation of these curves was reported elsewhere (Bracamonte et al., 2025)

**ESM2.4. Sources of experimental data for validation of untreated experimental MR in dogs.**

| **Experimental measurement** | **Sources** |
| --- | --- |
| **History of LV mass/BW fold change** | (Carabello et al., 1989b, 1992; Dell’Italia et al., 1995; Dell’italia et al., 1997; Hankes et al., 2006; Katayama et al., 1988; Kihara et al., 1988; Kleaveland et al., 1988; Matsuo et al., 1998; Nagatsu et al., 1994, 2000; Nakano et al., 1991; Nemoto et al., 2002, 2005; Pat et al., 2008, 2010; Perry et al., 2002; Sabri et al., 2008; Spinale et al., 1993; J. M. Stewart et al., 1992; Tallaj et al., 2003; Tsutsui et al., 1994; Urabe et al., 1992; Zheng et al., 2009) |
| **History of end diastolic volume** | (Berko et al., 1987; Carabello et al., 1989b, 1991; Dell’Italia et al., 1995; Dell’italia et al., 1997; Katayama et al., 1988; Kleaveland et al., 1988; Lee et al., 1985; Liu et al., 2013; Matsuo et al., 1998; Nakano et al., 1990, 1991; Nemoto et al., 2002, 2005; Pat et al., 2008, 2010; Perry et al., 2002; Spinale et al., 1993; J. A. Stewart et al., 2003; Trappanese et al., 2015; Tsutsui et al., 1994; Urabe et al., 1992; Zheng et al., 2009) |
| **History of end systolic volume** | (Berko et al., 1987; Carabello et al., 1989b; Dell’Italia et al., 1995; Dell’italia et al., 1997; Katayama et al., 1988; Kleaveland et al., 1988; Lee et al., 1985; Liu et al., 2013; Pat et al., 2008, 2010; Perry et al., 2002; Trappanese et al., 2015; Zheng et al., 2009) |
| **History of ejection fraction** | (Berko et al., 1987; Carabello et al., 1992; Dell’Italia et al., 1995; Dell’italia et al., 1997; Katayama et al., 1988; Kleaveland et al., 1988; Matsuo et al., 1998; Nagatsu et al., 1994, 2000; Nakano et al., 1991; Nemoto et al., 2002, 2005; Pat et al., 2010; Perry et al., 2002; Spinale et al., 1993; J. M. Stewart et al., 1992; Zheng et al., 2009) |
| **History of Mean Arterial Pressure** | (Hankes et al., 2006; Kleaveland et al., 1988; Perry et al., 2002; Sabri et al., 2008) |
| **History of Mean Pulmonary Arterial Pressure** | (Dell’Italia et al., 1995; Dell’italia et al., 1997; Kleaveland et al., 1988; Pat et al., 2008, 2010; Perry et al., 2002; J. M. Stewart et al., 1992; Tallaj et al., 2003) |

# References

Abassi, Z., Goltsman, I., Karram, T., Winaver, J., & Hoffman, A. (2011). Aortocaval Fistula in Rat: A Unique Model of Volume‐Overload Congestive Heart Failure and Cardiac Hypertrophy. *BioMed Research International*, *2011*(1). https://doi.org/10.1155/2011/729497

Arnal, J. F., Philippe, M., Laboulandine, I., & Michel, J. B. (1993). Effect of perindopril in rat cardiac volume overload. *American Heart Journal*, *126*(3), 776–782. https://doi.org/10.1016/0002-8703(93)90929-4

Asano, K., Masuda, K., Okumura, M., Kadosawa, T., & Fujinaga, T. (1999). Plasma Atrial and Brain Natriuretic Peptide Levels in Dogs with Congestive Heart Failure. *Journal of Veterinary Medical Science*, *61*(5), 523–529. https://doi.org/10.1292/jvms.61.523

Asano, K., Masuda, K., Okumura, M., Kadosawa, T., & Fujinaga, T. (2001). Association between Exogenous Atrial Natriuretic Peptide and Hemodynamics in Dogs with Congestive Heart Failure Produced by Experimental Mitral Regurgitation. *Journal of Veterinary Medical Science*, *63*(3), 243–250. https://doi.org/10.1292/JVMS.63.243

Bauer, P., Regitz-Zagrosek, V., Kallisch, H., Linz, W., Schoelkens, B., Hildebrandt, A. G., & Fleck, E. (1997). Myocardial angiotensin receptor type 1 gene expression in a rat model of cardiac volume overload. *Basic Research in Cardiology*, *92*(3), 139–146. https://doi.org/10.1007/BF00788631/METRICS

Berko, B., Gaasch, W. H., Tanigawa, N., Smith, D., & Craige, E. (1987). Disparity between ejection and end-systolic indexes of left ventricular contractility in mitral regurgitation. *Circulation*, *75*(6), 1310–1319. https://doi.org/10.1161/01.CIR.75.6.1310

Bracamonte, J. H., Watkins, L., Pat, B., Dell’Italia, L. J., Saucerman, J. J., & Holmes, J. W. (2025). Contributions of mechanical loading and hormonal changes to eccentric hypertrophy during volume overload: A Bayesian analysis using logic-based network models. *PLOS Computational Biology*, *21*(4), e1012390. https://doi.org/10.1371/JOURNAL.PCBI.1012390

Carabello, B. A., Nakano, K., Corin, W., Biederman, R., & Spann, J. F. (1989a). Left ventricular function in experimental volume overload hypertrophy. *The American Journal of Physiology*, *256*(4 Pt 2), H974-81. https://doi.org/10.1152/ajpheart.1989.256.4.H974

Carabello, B. A., Nakano, K., Corin, W., Biederman, R., & Spann, J. F. (1989b). Left ventricular function in experimental volume overload hypertrophy. *American Journal of Physiology-Heart and Circulatory Physiology*, *256*(4), H974–H981. https://doi.org/10.1152/ajpheart.1989.256.4.H974

Carabello, B. A., Nakano, K., Ishihara, K., Kanazawa, S., W Biederman, R. W., & Spann Jr, J. F. (1991). Coronary blood flow in dogs with contractile dysfunction due to experimental volume overload. *Circulation*, *83*(3), 1063–1075. https://doi.org/10.1161/01.CIR.83.3.1063

Carabello, B. A., Zile, M. R., Tanaka, R., & Cooper IV, G. (1992). Left ventricular hypertrophy due to volume overload versus pressure overload. *Https://Doi.Org/10.1152/Ajpheart.1992.263.4.H1137*, *263*(4 32-4), H1137–H1144. https://doi.org/10.1152/AJPHEART.1992.263.4.H1137

Cavero, P. G., Miller, W. L., Heublein, D. M., Margulies, K. B., & Burnett, J. C. (1990). Endothelin in experimental congestive heart failure in the anesthetized dog. *American Journal of Physiology-Renal Physiology*, *259*(2), F312–F317. https://doi.org/10.1152/ajprenal.1990.259.2.F312

Dell’italia, L. J., Balcells, E., Meng, Q. C., Su, X., Schultz, D., Bishop, S. P., Machida, N., Straeter-Knowlen, I. M., Hankes, G. H., Dillon, R., Cartee, R. E., & Oparil, S. (1997). Volume-overload cardiac hypertrophy is unaffected by ACE inhibitor treatment in dogs. *American Journal of Physiology-Heart and Circulatory Physiology*, *273*(2), H961–H970. https://doi.org/10.1152/ajpheart.1997.273.2.H961

Dell’Italia, L. J., Meng, Q. C., Balcells, E., Straeter-Knowlen, I. M., Hankes, G. H., Dillon, R., Cartee, R. E., Orr, R., Bishop, S. P., Oparil, S., & et, al. (1995). Increased ACE and chymase-like activity in cardiac tissue of dogs with chronic mitral regurgitation. *American Journal of Physiology-Heart and Circulatory Physiology*, *269*(6), H2065–H2073. https://doi.org/10.1152/ajpheart.1995.269.6.H2065

Dent, M. R., Das, S., & Dhalla, N. S. (2007). Alterations in both death and survival signals for apoptosis in heart failure due to volume overload. *Journal of Molecular and Cellular Cardiology*, *43*(6), 726–732. https://doi.org/10.1016/J.YJMCC.2007.09.001

Du, Y., Plante, E., Janicki, J. S., & Brower, G. L. (2010). Temporal Evaluation of Cardiac Myocyte Hypertrophy and Hyperplasia in Male Rats Secondary to Chronic Volume Overload. *The American Journal of Pathology*, *177*(3), 1155–1163. https://doi.org/10.2353/ajpath.2010.090587

Fareh, J., Touyz, R. M., Schiffrin, E. L., & Thibault, G. (1996). Endothelin-1 and Angiotensin II Receptors in Cells From Rat Hypertrophied Heart. *Circulation Research*, *78*(2), 302–311. https://doi.org/10.1161/01.RES.78.2.302

Fomovsky, G. M., Clark, S. A., Parker, K. M., Ailawadi, G., & Holmes, J. W. (2012). Anisotropic reinforcement of acute anteroapical infarcts improves pump function. *Circulation: Heart Failure*, *5*(4), 515–522. https://doi.org/10.1161/CIRCHEARTFAILURE.111.965731

Freire, G., Ocampo, C., Ilbawi, N., Griffin, A. J., & Gupta, M. (2007). Overt expression of AP-1 reduces alpha myosin heavy chain expression and contributes to heart failure from chronic volume overload. *Journal of Molecular and Cellular Cardiology*, *43*(4), 465–478. https://doi.org/10.1016/J.YJMCC.2007.07.046

Gardner, J. D., Murray, D. B., Voloshenyuk, T. G., Brower, G. L., Bradley, J. M., & Janicki, J. S. (2010). Estrogen attenuates chronic volume overload induced structural and functional remodeling in male rat hearts. *American Journal of Physiology-Heart and Circulatory Physiology*, *298*(2), H497–H504. https://doi.org/10.1152/ajpheart.00336.2009

Hankes, G. H., Ardell, J. L., Tallaj, J., Wei, C.-C., Aban, I., Holland, M., Rynders, P., Dillon, R., Cardinal, R., Hoover, D. B., Armour, J. A., Husain, A., & Dell’Italia, L. J. (2006). β _1_ <scp>-</scp> Adrenoceptor blockade mitigates excessive norepinephrine release into cardiac interstitium in mitral regurgitation in dog. *American Journal of Physiology-Heart and Circulatory Physiology*, *291*(1), H147–H151. https://doi.org/10.1152/ajpheart.00951.2005

Hori, Y., Sano, N., Kanai, K., Hoshi, F., Itoh, N., & Higuchi, S. I. (2010). Acute cardiac volume load-related changes in plasma atrial natriuretic peptide and N-terminal pro-B-type natriuretic peptide concentrations in healthy dogs. *The Veterinary Journal*, *185*(3), 317–321. https://doi.org/10.1016/J.TVJL.2009.06.008

Huang, M., Hester, R. L., & Guyton, A. C. (1992). Hemodynamic changes in rats after opening an arteriovenous fistula. *Https://Doi.Org/10.1152/Ajpheart.1992.262.3.H846*, *262*(3 31-3). https://doi.org/10.1152/AJPHEART.1992.262.3.H846

Ichiki, T., Boerrigter, G., Huntley, B. K., Sangaralingham, S. J., McKie, P. M., Harty, G. J., Harders, G. E., & Burnett, J. C. (2013). Differential expression of the pro-natriuretic peptide convertases corin and furin in experimental heart failure and atrial fibrosis. *American Journal of Physiology-Regulatory, Integrative and Comparative Physiology*, *304*(2), R102–R109. https://doi.org/10.1152/ajpregu.00233.2012

Imamura, T., McDermott, P. J., Kent, R. L., Nagatsu, M., Cooper IV, G., & Carabello, B. A. (1994). Acute changes in myosin heavy chain synthesis rate in pressure versus volume overload. *Circulation Research*, *75*(3), 418–425. https://doi.org/10.1161/01.RES.75.3.418

Ishiye, M., Umemura, K., Uematsu, T., & Nakashima, M. (1995). Effects of Losartan, an Angiotensin II Antagonist, on the Development of Cardiac Hypertrophy Due to Volume Overload. *Biological and Pharmaceutical Bulletin*, *18*(5), 700–704. https://doi.org/10.1248/BPB.18.700

Jobe, L. J., Meléndez, G. C., Levick, S. P., Du, Y., Brower, G. L., & Janicki, J. S. (2009). TNF-α inhibition attenuates adverse myocardial remodeling in a rat model of volume overload. *American Journal of Physiology-Heart and Circulatory Physiology*, *297*(4), H1462–H1468. https://doi.org/10.1152/ajpheart.00442.2009

Katayama, K., Tajimi, T., Guth, B. D., Matsuzaki, M., Lee, J. D., Seitelberger, R., & Peterson, K. L. (1988). Early diastolic filling dynamics during experimental mitral regurgitation in the conscious dog. *Circulation*, *78*(2), 390–400. https://doi.org/10.1161/01.CIR.78.2.390

Kihara, Y., Sasayama, S., Miyazaki, S., Onodera, T., Susawa, T., Nakamura, Y., Fujiwara, H., & Kawai, C. (1988). Role of the left atrium in adaptation of the heart to chronic mitral regurgitation in conscious dogs. *Circulation Research*, *62*(3), 543–553. https://doi.org/10.1161/01.RES.62.3.543

Kleaveland, J. P., Kussmaul, W. G., Vinciguerra, T., Diters, R., & Carabello, B. A. (1988). Volume overload hypertrophy in a closed-chest model of mitral regurgitation. *American Journal of Physiology-Heart and Circulatory Physiology*, *254*(6), H1034–H1041. https://doi.org/10.1152/ajpheart.1988.254.6.H1034

Kolpakov, M. A., Seqqat, R., Rafiq, K., Xi, H., Margulies, K. B., Libonati, J. R., Powel, P., Houser, S. R., Dell’italia, L. J., & Sabri, A. (2009). Pleiotropic effects of neutrophils on myocyte apoptosis and left ventricular remodeling during early volume overload. *Journal of Molecular and Cellular Cardiology*, *47*(5), 634–645. https://doi.org/10.1016/J.YJMCC.2009.08.016

Kristen, A. V., Just, A., Haass, M., & Seller, H. (2002). Central hypercapnic chemoreflex modulation of renal sympathetic nerve activity in experimental heart failure. *Basic Research in Cardiology*, *97*(2), 177–186. https://doi.org/10.1007/S003950200009/METRICS

Kristen, A. V., Kreusser, M. M., Lehmann, L., Kinscherf, R., Katus, H. A., Haass, M., & Backs, J. (2006). Preserved Norepinephrine Reuptake but Reduced Sympathetic Nerve Endings in Hypertrophic Volume-Overloaded Rat Hearts. *Journal of Cardiac Failure*, *12*(7), 577–583. https://doi.org/10.1016/J.CARDFAIL.2006.05.006

Lachance, D., Dhahri, W., Drolet, M.-C., Roussel, É., Gascon, S., Sarrhini, O., Rousseau, J. A., Lecomte, R., Arsenault, M., & Couet, J. (2014). Endurance training or beta-blockade can partially block the energy metabolism remodeling taking place in experimental chronic left ventricle volume overload. *BMC Cardiovascular Disorders*, *14*(1), 190. https://doi.org/10.1186/1471-2261-14-190

Langenickel, T., Pagel, I., Höhnel, K., Dietz, R., & Willenbrock, R. (2000). Differential regulation of cardiac ANP and BNP mRNA in different stages of experimental heart failure. *American Journal of Physiology-Heart and Circulatory Physiology*, *278*(5), H1500–H1506. https://doi.org/10.1152/ajpheart.2000.278.5.H1500

Lee, J. D., Sasayama, S., Kihara, Y., Ohyagi, A., Fujisawa, A., Yui, Y., & Kawai, C. (1985). Adaptations of the left ventricle to chronic volume overload induced by mitral regurgitation in conscious dogs. *Heart and Vessels*, *1*(1), 9–15. https://doi.org/10.1007/BF02066481/METRICS

Liu, Y., Dillon, A. R., Tillson, M., Makarewich, C., Nguyen, V., Dell’Italia, L., Sabri, A. K., Rizzo, V., & Tsai, E. J. (2013). Volume overload induces differential spatiotemporal regulation of myocardial soluble guanylyl cyclase in eccentric hypertrophy and heart failure. *Journal of Molecular and Cellular Cardiology*, *60*(1), 72–83. https://doi.org/10.1016/J.YJMCC.2013.03.019

Matsuo, T., Carabello, B. A., Nagatomo, Y., Koide, M., Hamawaki, M., Zile, M. R., & McDermott, P. J. (1998). Mechanisms of cardiac hypertrophy in canine volume overload. *American Journal of Physiology-Heart and Circulatory Physiology*, *275*(1), H65–H74. https://doi.org/10.1152/ajpheart.1998.275.1.H65

Nagatsu, M., Spinale, F. G., Koide, M., Tagawa, H., DeFreitas, G., Cooper, G., & Carabello, B. A. (2000). Bradycardia and the role of beta-blockade in the amelioration of left ventricular dysfunction. *Circulation*, *101*(6), 653–659. https://doi.org/10.1161/01.cir.101.6.653

Nagatsu, M., Zile, M. R., Tsutsui, H., Schmid, P. G., DeFreyte, G., Cooper IV, G., & Carabello, B. A. (1994). Native beta-adrenergic support for left ventricular dysfunction in experimental mitral regurgitation normalizes indexes of pump and contractile function. *Circulation*, *89*(2), 818–826. https://doi.org/10.1161/01.CIR.89.2.818

Nakano, K., Sugawara, M., Ishihara, K., Kanazawa, S., Corin, W. J., Denslow, S., Biederman, R. W. W., & Carabello, B. A. (1990). Myocardial stiffness derived from end-systolic wall stress and logarithm of reciprocal of wall thickness. Contractility index independent of ventricular size. *Circulation*, *82*(4), 1352–1361. https://doi.org/10.1161/01.CIR.82.4.1352

Nakano, K., Swindle, M. M., Spinale, F., Ishihara, K., Kanazawa, S., Smith, A., Biederman, R. W., Clamp, L., Hamada, Y., & Zile, M. R. (1991). Depressed contractile function due to canine mitral regurgitation improves after correction of the volume overload. *Journal of Clinical Investigation*, *87*(6), 2077–2086. https://doi.org/10.1172/JCI115238

Nemoto, S., Hamawaki, M., De Freitas, G., & Carabello, B. A. (2002). differential effects of the angiotensin-converting enzyme inhibitor lisinopril versus the beta-adrenergic receptor blocker atenolol on hemodynamics and left ventricular contractile function in experimental mitral regurgitation. *Journal of the American College of Cardiology*, *40*(1), 149–154. https://doi.org/10.1016/S0735-1097(02)01926-5

Nemoto, S., Razeghi, P., Ishiyama, M., De Freitas, G., Taegtmeyer, H., & Carabello, B. A. (2005). PPAR-γ agonist rosiglitazone ameliorates ventricular dysfunction in experimental chronic mitral regurgitation. *American Journal of Physiology-Heart and Circulatory Physiology*, *288*(1), H77–H82. https://doi.org/10.1152/ajpheart.01246.2003

Oka, T., Nishimura, H., Ueyama, M., Kubota, J., & Kawamura, K. (1993). Lisinopril reduces cardiac hypertrophy and mortality in rats with aortocaval fistula. *European Journal of Pharmacology*, *234*(1), 55–60. https://doi.org/10.1016/0014-2999(93)90705-M

Pagel, I., Langenickel, T., Höhnel, K., Philipp, S., Nüssler, A. K., Blum, W. F., Aubert, M. L., Dietz, R., & Willenbrock, R. (2002). Cardiac and renal effects of growth hormone in volume overload-induced heart failure: role of NO. *Hypertension (Dallas, Tex. : 1979)*, *39*(1), 57–62. https://doi.org/10.1161/HY0102.098323

Pat, B., Chen, Y., Killingsworth, C., Gladden, J. D., Shi, K., Zheng, J., Powell, P. C., Walcott, G., Ahmed, M. I., Gupta, H., Desai, R., Wei, C. C., Hase, N., Kobayashi, T., Sabri, A., Granzier, H., Denney, T., Tillson, M., Dillon, A. R., … Dell’Italia, L. J. (2010). Chymase inhibition prevents fibronectin and myofibrillar loss and improves cardiomyocyte function and LV torsion angle in dogs with isolated mitral regurgitation. *Circulation*, *122*(15), 1488–1495. https://doi.org/10.1161/CIRCULATIONAHA.109.921619

Pat, B., Killingsworth, C., Denney, T., Zheng, J., Powell, P., Tillson, M., Dillon, A. R., & Dell’Italia, L. J. (2008). Dissociation between cardiomyocyte function and remodeling with β-adrenergic receptor blockade in isolated canine mitral regurgitation. *American Journal of Physiology-Heart and Circulatory Physiology*, *295*(6), H2321–H2327. https://doi.org/10.1152/ajpheart.00746.2008

Perry, G. J., Wei, C. C., Hankes, G. H., Dillon, S. R., Rynders, P., Mukherjee, R., Spinale, F. G., & Dell’Italia, L. J. (2002). Angiotensin II receptor blockade does not improve left ventricular function and remodeling in subacute mitral regurgitation in the dog. *Journal of the American College of Cardiology*, *39*(8), 1374–1379. https://doi.org/10.1016/S0735-1097(02)01763-1

Prasad, K., Khatter, J. C., & Bharadwaj, B. (1977). Cardiovascular Function in Experimental Chronic Mitral Insufficiency in Dogs. *Japanese Heart Journal*, *18*(6), 823–840. https://doi.org/10.1536/IHJ.18.823

Pu, M., Gao, Z., Pu, D. K., & Davidson, W. R. (2013). Effects of early, late, and long-term nonselective β-blockade on left ventricular remodeling, function, and survival in chronic organic mitral regurgitation. *Circulation: Heart Failure*, *6*(4), 756–762. https://doi.org/10.1161/CIRCHEARTFAILURE.112.000196

Pu, M., Gao, Z., Zhang, X., Liao, D., Pu, D. K., Brennan, T., & Davidson, W. R. (2009). Impact of mitral regurgitation on left ventricular anatomic and molecular remodeling and systolic function: implication for outcome. *American Journal of Physiology-Heart and Circulatory Physiology*, *296*(6), H1727–H1732. https://doi.org/10.1152/ajpheart.00882.2008

Ray, L., Mathieu, M., Jespers, P., Hadad, I., Mahmoudabady, M., Pensis, A., Motte, S., Peters, I. R., Naeije, R., & McEntee, K. (2008). Early increase in pulmonary vascular reactivity with overexpression of endothelin-1 and vascular endothelial growth factor in canine experimental heart failure. *Experimental Physiology*, *93*(3), 434–442. https://doi.org/10.1113/EXPPHYSIOL.2007.040469

Ross, J., & McCullagh, W. H. (1972). Nature of Enhanced Performance of the Dilated Left Ventricle in the Dog during Chronic Volume Overloading. *Circulation Research*, *30*(5), 549–556. https://doi.org/10.1161/01.RES.30.5.549

Ross, J., Sonnenblick, E. H., Taylor, R. R., Spotnitz, H. M., & Covell, J. W. (1971). Diastolic Geometry and Sarcomere Lengths in the Chronically Dilated Canine Left Ventricle. *Circulation Research*, *28*(1), 49–61. https://doi.org/10.1161/01.RES.28.1.49

Sabri, A., Rafiq, K., Seqqat, R., Kolpakov, M. A., Dillon, R., & Dell’italia, L. J. (2008). Sympathetic Activation Causes Focal Adhesion Signaling Alteration in Early Compensated Volume Overload Attributable to Isolated Mitral Regurgitation in the Dog. *Circulation Research*, *102*(9), 1127–1136. https://doi.org/10.1161/CIRCRESAHA.107.163642

Seqqat, R., Guo, X., Rafiq, K., Kolpakov, M. A., Guo, J., Koch, W. J., Houser, S. R., Dell’italia, L. J., & Sabri, A. (2012). Beta1-adrenergic receptors promote focal adhesion signaling downregulation and myocyte apoptosis in acute volume overload. *Journal of Molecular and Cellular Cardiology*, *53*(2), 240–249. https://doi.org/10.1016/j.yjmcc.2012.05.004

Shuji, H., Takuroh, I., Takeshi, M., Yuichiro, I., Johji, K., Kazuo, K., Yasushi, K., & Tanenao, E. (2000). Differential responses of circulating and tissue adrenomedullin and gene expression to volume overload. *Journal of Cardiac Failure*, *6*(2), 120–129. https://doi.org/10.1016/S1071-9164(00)90014-9

Spinale, F. G., Ishihra, K., Zile, M., DeFryte, G., Crawford, F. A., & Carabello, B. A. (1993). Structural basis for changes in left ventricular function and geometry because of chronic mitral regurgitation and after correction of volume overload. *The Journal of Thoracic and Cardiovascular Surgery*, *106*(6), 1147–1157. https://doi.org/10.1016/S0022-5223(19)33992-3

Spotnitz, H. M., Sonnenblick, E. H., & Spiro, D. (1966). Relation of Ultrastructure to Function in the Intact Heart: Sarcomere Structure Relative to Pressure Volume Curves of Intact Left Ventricles of Dog and Cat. *Circulation Research*, *18*(1), 49–66. https://doi.org/10.1161/01.RES.18.1.49

Stewart, J. A., Wei, C. C., Brower, G. L., Rynders, P. E., Hankes, G. H., Dillon, A. R., Lucchesi, P. A., Janicki, J. S., & Dell’Italia, L. J. (2003). Cardiac mast cell- and chymase-mediated matrix metalloproteinase activity and left ventricular remodeling in mitral regurgitation in the dog. *Journal of Molecular and Cellular Cardiology*, *35*(3), 311–319. https://doi.org/10.1016/S0022-2828(03)00013-0

Stewart, J. M., Patel, M. B., Wang, J., Ochoa, M., Gewitz, M., Loud, A. V., Anversa, P., & Hintze, T. H. (1992). Chronic elevation of norepinephrine in conscious dogs produces hypertrophy with no loss of LV reserve. *American Journal of Physiology-Heart and Circulatory Physiology*, *262*(2), H331–H339. https://doi.org/10.1152/ajpheart.1992.262.2.H331

Suzuki, H., Maehara, K., Yaoita, H., & Maruyama, Y. (2004). Altered Effects of Angiotensin II Type 1 and Type 2 Receptor Blockers on Cardiac Norepinephrine Release and Inotropic Responses During Cardiac Sympathetic Nerve Stimulation in Aorto-Caval Shunt Rats. *Circulation Journal*, *68*(7), 683–690. https://doi.org/10.1253/CIRCJ.68.683

Tallaj, J., Wei, C.-C., Hankes, G. H., Holland, M., Rynders, P., Dillon, A. R., Ardell, J. L., Armour, J. A., Lucchesi, P. A., & Dell’Italia, L. J. (2003). β1-Adrenergic Receptor Blockade Attenuates Angiotensin II–Mediated Catecholamine Release Into the Cardiac Interstitium in Mitral Regurgitation. *Circulation*, *108*(2), 225–230. https://doi.org/10.1161/01.CIR.0000079226.48637.5A

Trappanese, D. M., Liu, Y., McCormick, R. C., Cannavo, A., Nanayakkara, G., Baskharoun, M. M., Jarrett, H., Woitek, F. J., Tillson, D. M., Dillon, A. R., Recchia, F. A., Balligand, J.-L., Houser, S. R., Koch, W. J., Dell’Italia, L. J., & Tsai, E. J. (2015). Chronic β1-adrenergic blockade enhances myocardial β3-adrenergic coupling with nitric oxide-cGMP signaling in a canine model of chronic volume overload: new insight into mechanisms of cardiac benefit with selective β1-blocker therapy. *Basic Research in Cardiology*, *110*(1), 456. https://doi.org/10.1007/s00395-014-0456-3

Tsutsui, H., Spinale, F. G., Nagatsu, M., Schmid, P. G., Ishihara, K., DeFreyte, G., Cooper, G., & Carabello, B. A. (1994). Effects of chronic beta-adrenergic blockade on the left ventricular and cardiocyte abnormalities of chronic canine mitral regurgitation. *Journal of Clinical Investigation*, *93*(6), 2639–2648. https://doi.org/10.1172/JCI117277

Urabe, Y., Mann, D. L., Kent, R. L., Nakano, K., Tomanek, R. J., Carabello, B. A., & Cooper IV, G. (1992). Cellular and ventricular contractile dysfunction in experimental canine mitral regurgitation. *Circulation Research*, *70*(1), 131–147. https://doi.org/10.1161/01.RES.70.1.131

Wang, X. H., Zhuo, X. Z., Ni, Y. J., Gong, M., Wang, T. Z., Lu, Q., & Ma, A. Q. (2012). Improvement of cardiac function and reversal of gap junction remodeling by Neuregulin-1β in volume-overloaded rats with heart failure. *Journal of Geriatric Cardiology : JGC*, *9*(2), 172. https://doi.org/10.3724/SP.J.1263.2012.03271

Willenbrock, R., Stauss, H., Scheuermann, M., Osterziel, K. J., Unger, T., & Dietz, R. (1997). Effect of chronic volume overload on baroreflex control of heart rate and sympathetic nerve activity. *The American Journal of Physiology*, *273*(6), H2580-5. https://doi.org/10.1152/ajpheart.1997.273.6.H2580

Wojciechowski, P., Juric, D., Louis, X. L., Thandapilly, S. J., Yu, L., Taylor, C., & Netticadan, T. (2010). Resveratrol Arrests and Regresses the Development of Pressure Overload- but Not Volume Overload-Induced Cardiac Hypertrophy in Rats. *The Journal of Nutrition*, *140*(5), 962–968. https://doi.org/10.3945/JN.109.115006

Yamakawa, H., Imamura, T., Matsuo, T., Onitsuka, H., Tsumori, Y., Kato, J., Kitamura, K., Koiwaya, Y., & Eto, T. (2000). Diastolic wall stress and ANG II in cardiac hypertrophy and gene expression induced by volume overload. *American Journal of Physiology-Heart and Circulatory Physiology*, *279*(6), H2939–H2946. https://doi.org/10.1152/ajpheart.2000.279.6.H2939

Zhang, W., Elimban, V., Xu, Y. J., Zhang, M., Nijjar, M. S., & Dhalla, N. S. (2010). Alterations of Cardiac ERK1/2 Expression and Activity Due to Volume Overload Were Attenuated by the Blockade of RAS. *Journal of Cardiovascular Pharmacology and Therapeutics*, *15*(1), 84–92. https://doi.org/10.1177/1074248409356430

Zheng, J., Chen, Y., Pat, B., Dell’Italia, D. L. A., Tillson, M., Dillon, A. R., Powell, P. C., Shi, K., Shah, N., Denney, T., Husain, A., & Dell’Italia, L. J. (2009). Molecular cardiology microarray identifies extensive downregulation of noncollagen extracellular matrix and profibrotic growth factor genes in chronic isolated mitral regurgitation in the dog. *Circulation*, *119*(15), 2086–2095. https://doi.org/10.1161/CIRCULATIONAHA.108.826230
