# Supplementary material for: Multiscale model calibrated with a Bayesian algorithm predicts long-term outcomes of treatments for mitral valve regurgitation in animal models
Source: Biomech Model Mechanobiol. 2026 Sep 20;25(5):107. doi: 10.1007/s10237-026-02123-0 (PMC13590452; doi:10.1007/s10237-026-02123-0)
Supplement: Supplementary file 2 [file 10237_2026_2123_MOESM3_ESM.docx]

**Multiscale model calibrated with a Bayesian algorithm predicts long-term outcomes of treatments for mitral valve regurgitation in animal models**

Johane H. Bracamonte, Lionel Watkins, Jeffrey J. Saucerman, Jeffrey W. Holmes

**Supplementary Material ESM3. Markov Chain Monte Carlo Calibration.**

**ESM3.1. MCMC Calibration Algorithm**

The MCMC is a random walk algorithm that iteratively solves the numerical model with random selections of input parameters x_i_ picked from a prescribed probability distribution of the parameter space known as the prior probability distribution function (prior PDF). On each iteration, we evaluated the likelihood of the model’s outputs against the probability distribution of the experimental datasets assigned for calibration. In MCMC, the acceptance criteria of the current parameter set depends only on the previous iteration (x_i-1_) (Hastings, 1970).

For the acceptance criteria we applied a special case of the Metropolis-Hastings algorithm. In its standard form, the acceptance probability (AP) of a parameter is calculated as the product of the posterior probability density ratio, and the proposal probability ratio, such as:

| $AP\left( x_{i},x_{i-1} \right)=\min\left( 1,\frac{\Pi\left( x_{i} \right)}{\Pi\left( x_{i-1} \right)}\frac{g\left( x_{i}, x_{i-1} \right)}{g\left( x_{i-1},x_{i} \right)} \right).$ | (ESM3.1) |
| --- | --- |

In this work we assumed the proposal function to be Gaussian and symmetric, that is, $g\left( x_{i}, x_{i-1} \right)= g\left( x_{i-1},x_{i} \right)$, and assumed the likelihood of a parameter set $f(x_{i})$ to be proportional to $\Pi\left( x_{i} \right)$, so that

| $AP\left( x_{i},x_{i-1} \right)=\min\left( 1,\frac{f\left( x_{i} \right)}{f(x_{i-1)}} \right)$ | (ESM3.2) |
| --- | --- |

a simplified form sometimes called the Metropolis algorithm (Andrieu et al., 2003).

The Gibbs sampling algorithm is a convenient method for the exploration of high-dimensional probability distributions and consists of updating on each MCMC iteration only one parameter at a time while keeping the rest of the parameters fixed at the previous iteration values.

**ESM3.2. Computational implementation of the MCMC algorithm.**

We programmed a custom MCMC algorithm in MATLAB. We defined prior probability distribution functions as Gaussian or normal functions based on previously acquired experience with the models, including the extensive parameter sensitivity analysis by Witzenburg and Holmes for the biomechanics model, and by Bracamonte et al. for the cardiomyocyte network model (Bracamonte et al., 2025; Witzenburg & Holmes, 2018, 2019). Sampling from the prior distributions was done using the built-in MATLAB function random:

| $x_{j}=random\left( 'Normal^{'},m,s \right)$, | (ESM3.3) |
| --- | --- |

where ‘Normal’ indicates we assumed the prior distribution of each parameter was normally distributed around mean m and standard deviation s.

We estimated the likelihood of each output with respect to corresponding experimental data using the built-in MATLAB function pdf,

| $f_{j}=pdf\left( 'Normal^{'},y_{j},\mu,\sigma\right)$, | (ESM3.4) |
| --- | --- |

where, ‘Normal’ refers to the assumption that all experimental data is distributed normally around a mean μ and a standard deviation σ. The overall likelihood of a parameter set was calculated as the product of all output likelihoods.

| $f_{total}=\prod_{j=1}^{Noutputs} f_{j}$. | (ESM3.5) |
| --- | --- |

The likelihood of the iteration was then used to compute the Metropolis-Hastings acceptance criteria AP according to equation ESM3.2 above.

To implement the acceptance algorithm, on each MCMC iteration we generated a random number $u$ in the [0-1] range and compared it to the AP. If $u\leq AP$ we accept the new parameter set $x_{i}$ and save it, if $u>AP,$ we reject the new state x_i_, and save the previous state x_i-1_ instead. After sufficient iterations, the collection of saved parameter sets converged to a new probability distribution of the parameter space, or posterior PDF (Dhamala et al., 2018; Efendiev et al., 2006).

Prescribed variables were left fixed for six Gibbs sampling rounds before adopting new randomly generated values. This allowed us to consider a literature-derived distribution of values for prescribed variables like HR while still allowing the algorithm to reach convergence.

We verified the burn-in phase and convergence of the MCMC algorithm by calculating the mean and standard deviation of the log(likelyhood) and calibrated parameters on the last 5000 iterations. We defined the burn-in phase as the collection of initial iterations where the 5000-iteration means showed visible steady changes. We considered the process converged when the change in mean and standard deviation was below 1% for all parameters (Figure ESM3.1). We applied two different MCMC processes to calibrate the cardiovascular biomechanics model and the cardiomyocyte network model separately. We also calculated the mean and standard deviation of the log(likelihood) and calibrated parameters starting at the end of the burn-in phase. We verified the cumulated mean and standard deviation converged to the same values as those calculated on the last 5000 iterations (Figure ESM3.2 and ESM3.4).

Figure ESM3.1. Algorithm to apply convergence criteria for the MCMC algorithm.

**ESM3.3. Calibration of the Cardiomyocyte Network model.**

In previous work we calibrated a stand-alone version of the cardiomyocyte network model using a data-driven estimate of mechanical stimulation (myoStrain) (Bracamonte et al., 2025). In this work we perform a second calibration of the network model using mechanical strains derived from the biomechanics component of the multiscale model. For this network recalibration we followed the same methods as described in our 2025 paper. This time, however, we used the probability distributions for network parameters resulting from our previous study as the prior probability distribution functions (instead of a naïve approach). The prior PDFs were assumed to be $w_{back}=0.031\pm0.022$, $w_{ANGII}=0.010\pm0.002$, $w_{NE}=0.033\pm0.014$, and $w_{ET1}=0.056\pm0.025$.

We ran the MCMC for 40,000 iterations until reaching convergence. We identified a burn-in stage of 15,000 iterations, characterized by a steady increase in likelihood, and adjustment of the mean and range of network parameters. The acceptance rate of the MCMC algorithm for this model and parameter space was 33.48% (Figure ESM3.2).

The Cardiomyocyte Network model was calibrated using data from diverse animal models and experimental groups (37 studies of experimental MR in dogs and 33 research articles on volume overload in rats) reporting biomolecular changes across the history of volume overload, which may explain the relatively low mean likelihood of the model predictions and frequent acceptance of low-likelihood parameter sets (Bracamonte et al., 2025).

**Figure ESM3.2.** MCMC chain of log-likelihood evaluation for the cardiomyocyte network model. We identified a burn-in stage of 15000 iterations, during which the mean values of likelihood and model parameters changed steadily. Gray lines represent the chain of iteration likelihoods, read markers and error bars indicate mean and standard deviation of the previous 5000 iterations. Purple solid line represents the cumulative mean estimated from the end of the burn-in stage, and dashed purple lines indicate cumulative standard deviation.

**Figure ESM3.3.** Pyramid plot of MCMC accepted samples after burn-in stage. We found no significant correlation between calibrated parameters.

**ESM3.4. Calibration of the Cardiovascular Biomechanics model.**

The cardiovascular biomechanics model employed in this work has been used previously to reproduce both pressure and volume overload in dogs, with parameters being estimated via conventional optimization techniques to minimize the error between model outputs and experimental data. Furthermore, Witzenburg and Holmes performed a detailed parameter sensitivity analysis providing relevant insights on the parameter space of this model (Santamore & Burkhoff, 1991; Witzenburg & Holmes, 2018).

We used previously published parameter estimates to propose a prior probability distribution of the 11 parameters to be calibrated. The prior probability distributions were $A^{0}=2\pm1.5 mmHg$, $B^{0}=0.08\pm0.05 mL^{-1}$, $E^{0}=18\pm5 mmHg/mL$, $SBV^{0}=336\pm25$, $SBV^{1}=360\pm50$, $SVR^{0}=3.1\pm2.0$, $SVR^{1}=1.5\pm1.0$, $MVBR^{1}=0.3\pm0.1$, $PVR^{0}=0.5\pm0.3$, and $PVR^{1}=0.2\pm0.15$. We ran the MCMC algorithm for 60,000 iterations. Visual inspection reveals no evident burn-in stage, which suggests the selected prior PDFs were centered around a parameter set of high-likelihood (Figure ESM3.4).

**Figure ESM3.4.** MCMC chain of log-likelihood evaluation for the cardiovascular mechanics model. We identified a burn-in stage of 10000 iterations, during which changes in the range and mean values of likelihood and model parameters changed steadily. Gray lines represent the chain of iteration likelihoods, read markers and error bars indicate mean and standard deviation of the previous 5000 iterations. Purple solid line represents the cumulative mean estimated from the end of the burn-in stage, and dashed purple lines indicate cumulative standard deviation.

The acceptance rate was 4.8% for this MCMC. Typical causes for low acceptance rates include high-dimensionality of the parameter space, parameter correlation, large prior variance, or the effect of random variable updates. To compensate for a relatively low acceptance rate we ran the MCMC well beyond convergence to produce a reasonably large sample of the posterior. Attempts to improve acceptance rate by reducing the variance of the prior PDF and spacing out the update of random variables had little effect.

**Figure ESM3.5.** a) Pyramid plot of MCMC accepted samples after burn-in stage. b) We identified in the posterior sample correlations between parameters A and B, and parameters SVR and HR at baseline and acute states.

A correlation analysis of the posterior PDF reveals a strong correlation between parameters A and B (PCC=0.7, Figure ESM3.5a), which both modulate passive LV stiffness. There is also a strong correlation between SVR and the prescribed variable HR (PCC>0.5) (Figure ESM3.5b), which both modulate blood pressure. Thus, we suspect that parameter correlation is mainly responsible for the low acceptance rate of the Markov Chain.

**ESM3.5. Forward simulations of drug therapies.**

To complement the simulations shown in the manuscript on subsets of simulations selected to match the characteristics of the control groups in individual published studies, we also simulated chronic MR without and without drug therapies on the pool of 10,000 MR cases generated from the MCMC posterior PDF. In these simulations we assumed the drug therapy began with the induction of experimental MR and continued for 6 months. In these simulations we observed the same trends produced in the validation runs described in sections 2.7 and 3.2 of the main manuscript: chronic bB exacerbates LV hypertrophy and the other drugs display a slight protective effect with ARB having the largest influence (Figure ESM3.6). Notably, the variances of these MC simulations were larger than in the validation experiments shown in figure 5 of the main manuscript, which is consistent with the larger variance of the population (Figure 5a).

**Figure ESM3.6.**  Simulation of the effect of drugs on a MCMC posterior distribution sample of N=10,000 baseline conditions. Chronic bB exacerbates hypertrophy in chronic MR, while ARB, ACEi, and CMAi tend to prevent growth, with ARB being the most effective treatment.

**References**

Andrieu, C., De Freitas, N., Doucet, A., & Jordan, M. I. (2003). An introduction to MCMC for machine learning. *Machine Learning*, *50*(1–2), 5–43. https://doi.org/10.1023/A:1020281327116/METRICS

Bracamonte, J. H., Watkins, L., Pat, B., Dell’Italia, L. J., Saucerman, J. J., & Holmes, J. W. (2025). Contributions of mechanical loading and hormonal changes to eccentric hypertrophy during volume overload: A Bayesian analysis using logic-based network models. *PLOS Computational Biology*, *21*(4), e1012390. https://doi.org/10.1371/JOURNAL.PCBI.1012390

Dhamala, J., Arevalo, H. J., Sapp, J., Horácek, B. M., Wu, K. C., Trayanova, N. A., & Wang, L. (2018). Quantifying the uncertainty in model parameters using Gaussian process-based Markov chain Monte Carlo in cardiac electrophysiology. *Medical Image Analysis*, *48*, 43–57. https://doi.org/10.1016/J.MEDIA.2018.05.007

Efendiev, Y., Hou, T., & Luo, W. (2006). Preconditioning Markov Chain Monte Carlo Simulations Using Coarse-Scale Models. *SIAM Journal on Scientific Computing*, *28*(2), 776–803. https://doi.org/10.1137/050628568

Hastings, W. K. (1970). Monte Carlo sampling methods using Markov chains and their applications. *Biometrika*, *57*(1), 97–109. https://doi.org/10.1093/BIOMET/57.1.97

Santamore, W. P., & Burkhoff, D. (1991). Hemodynamic consequences of ventricular interaction as assessed by model analysis. *American Journal of Physiology-Heart and Circulatory Physiology*, *260*(1), H146–H157. https://doi.org/10.1152/ajpheart.1991.260.1.H146

Witzenburg, C. M., & Holmes, J. W. (2018). Predicting the Time Course of Ventricular Dilation and Thickening Using a Rapid Compartmental Model. *Journal of Cardiovascular Translational Research*, *11*(2), 109–122. https://doi.org/10.1007/S12265-018-9793-1

Witzenburg, C. M., & Holmes, J. W. (2019). The impact of hemodynamic reflex compensation following myocardial infarction on subsequent ventricular remodeling. *Journal of Biomechanical Engineering*, *141*(9). https://doi.org/10.1115/1.4043867/726864
